# Coordination-driven assembly of a ferrocene-functionalized lead iodide framework with enhanced stability and charge transfer for photocatalytic CO_2_-to-CH_3_OH conversion†

**DOI:** 10.1039/d4sc08216h

**Published:** 2025-03-31

**Authors:** Jinlin Yin, Yani He, Chen Sun, Yilin Jiang, Honghan Fei

**Affiliations:** a Shanghai Key Laboratory of Chemical Assessment and Sustainability, School of Chemical Science and Engineering, Tongji University 1239 Siping Rd. Shanghai 200092 China fei@tongji.edu.cn

## Abstract

Hybrid lead halides are promising photocatalysts due to their high structural tunability and excellent photophysical properties, but their ionic structures suffer from instability in polar environments and suppressed charge transfer between lead halide units and organic components. Herein, we successfully incorporated a ferrocene-based light-harvesting antenna into a lead iodide framework by coordination-driven assembly. The π-conjugated Pb^2+^-carboxylate linkage affords synergistic interactions between [Pb_2_I_2_]^2+^ chains and ferrocene linkers, achieving broad visible absorption up to 612.7 nm and efficient ligand-to-metal charge transfer for spatial charge separation. This ultrastable framework combines strong visible-light absorption of ferrocene centers with excellent charge transport of lead halide units, achieving 6e^−^ CO_2_ photoreduction to CH_3_OH coupled with ethanol oxidation. Mechanistic studies reveal that ferrocene photoexcitation followed by linker-to-metal charge transfer significantly enhances carrier accumulation, accelerating CH_3_O* intermediate formation as indicated by *in situ* spectroscopy and theoretical calculations.

## Abstract 2

Photocatalytic conversion of CO_2_ to CH_3_OH is achieved using a lead iodide framework. The π-conjugated coordination linkage between the ferrocene ligand and the lead iodide chains enhances structural stability and promotes charge separation.

## Graphical abstract

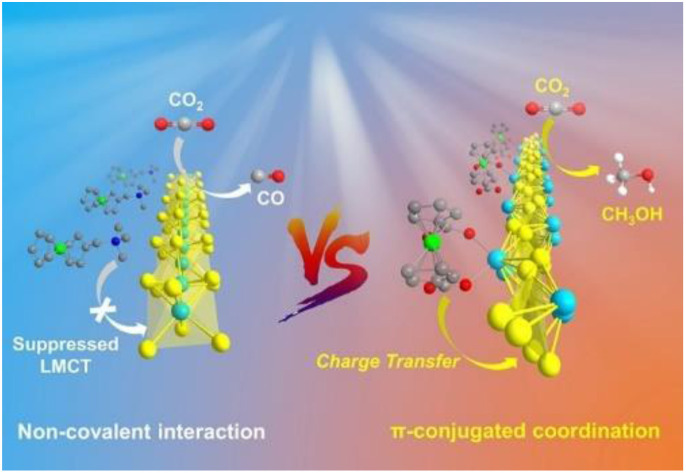

## Introduction

Chemical reduction of CO_2_ to methanol (CH_3_OH) is considered as the basis of the “methanol economy”, which is thermodynamically challenging and requires high energy input under industrially relevant conditions (*e.g.* high temperature and pressure).^1–3^ Mimicking the natural photosynthesis process, photocatalytic CO_2_ reduction is one of the promising approaches for realizing a carbon neutral methanol economy.^4–6^ However, selectivity control in CO_2_ photoreduction is extremely challenging, because the kinetically favorable 2e^−^ reduction products (*i.e.* CO and HCOOH) often have high energy requirements for further protonation.^7–12^ Compared to other C_1_ products, CH_3_OH is more demanding owing to its liquid state for easy storage and transportation as well as its role as a precursor to many commodity chemicals and biodiesel.^13–15^ Photocatalytic reduction of CO_2_ to CH_3_OH requires the simultaneous transfer of 6e^−^ and 6H^+^ to the semiconductor surface, suppressing the undesired electron–hole annihilation.^5,16^ Therefore, it is of key importance to improve carrier accumulation on the semiconductor to realize CO_2_-to-CH_3_OH transformation, and only a few studies have reported high selectivity using environmentally friendly reductants.^5,17,18^ For example, carbon nitride (CN) decorated with carbon dots (^m^CD) produces stoichiometric oxygen and CH_3_OH from water and CO_2_, which is largely ascribed to ^m^CD extracting holes from CN and preventing the surface adsorption of CH_3_OH.^16^

Lead halide hybrids are an emerging class of photocatalysts, spanning a variety of reactions including hydrogen production, CO_2_ reduction and organic transformation.^19–26^ This class of semiconductors exhibits intriguing photophysical properties, such as tunable bandgaps, high light-absorption coefficients and excellent carrier transport characteristics.^27–29^ However, the vast majority of them are susceptible to degradation upon exposure to high-polarity protic molecules, ascribed to their soft ionically bound lattices.^30,31^ Up to now, their photocatalytic applications in CO_2_ reduction are largely confined to low-polarity chemical conditions and limited to non-protic reducing products (*e.g.* CO and CH_4_).^32^

Our group focuses on the synthesis of lead halide frameworks based on carboxylate ligands as the organic component.^33^ Unlike conventional hybrid halides featuring ionic structures, our lead halide frameworks exhibit remarkable structural stability with excellent photophysical properties.^33–39^ For example, adipate-intercalated layered lead halide frameworks ([Pb_2_X_2_]^2+^[^−^O_2_C(CH_2_)_4_CO_2_^−^], X = F^−^/Cl^−^/Br^−^/I^−^) exhibit high chemical stability in aqueous solutions across a wide range of pH levels, as well as under aqueous boiling conditions.^33^ This synthetic strategy enables lead halide frameworks to achieve CO_2_ photoreduction to CO using water vapor as well as photocatalytic overall water splitting without any sacrificial agent.^40,41^ However, their photocatalytic efficiency remains limited due to the spatial separation of lead halide units by photochemically “inert” organocarboxylates, which often restricts visible-light absorption.^40,41^ Although unexplored in the literature, the integration of light-harvesting ferrocene-based linkers into lead iodide frameworks will be a promising photocatalysis platform, offering both strong photon absorption and enhanced carrier accumulation.

Ferrocene (Fc)-functionalized moieties exhibit outstanding visible-light absorption and charge transfer characteristics, making them excellent organometallic components in optoelectronic applications.^42,43^ However, there have been very few reports on Fc-functionalized lead halide hybrids, and the ionic bonding between lead halide units and Fc ligands hinders intrinsic electron transfer and effective charge separation for photocatalysis.^44,45^ Recently, Dong and colleagues demonstrated that grafting ferrocenecarboxylic acid (FCA) onto CsPbBr_3_ quantum dots substantially enhances photocatalytic efficiency for CO_2_-to-CO conversion, facilitating hot electron transfer from the FCA to the quantum dots.^46^ This improvement is attributed to the coordination bonds formed between these two components.^46^ However, the coordination-driven assembly of Fc-functionalized moieties and lead halide sublattices to afford an intrinsic framework-type structure remains an outstanding challenge, representing a significant advance for improving both light absorption and charge transfer in lead halide photocatalysis.

Herein, we report a robust lead iodide framework possessing 1D linear [Pb_2_I_2_]^2+^ chains, which are bridged by [Pb_2_O_2_] clusters into inorganic lamellar units. The inorganic sheets are coordinatively decorated with interlamellar 1,1′-ferrocene-dicarboxylates (Fcdc^2−^) as light-harvesting chromophores, stacked by van der Waals interactions. In contrast to Fc-based perovskite ((FMTMA)PbI_3_) with non-covalent interactions between the Fc moieties and lead halide building blocks, the formation of π-conjugated Pb^2+^-carboxylate coordination in our framework results in enhanced carrier delocalization and carrier transport. The material exhibits a broad visible-light wavelength coverage with an indirect semiconductive bandgap of ∼1.92 eV and efficient photoinduced charge transfer from Fcdc^2−^ to the 1D [Pb_2_I_2_]^2+^ chains. The favorable ligand-to-metal charge transfer (LMCT) process greatly improves photogenerated charge separation, as thoroughly investigated by surface photovoltage spectroscopy, Hall effect measurement and density functional theory (DFT) calculations. Combining the ultrastable nature and the intriguing carrier dynamics, the lead iodide framework achieves photoreduction of CO_2_ to CH_3_OH with exceptionally high selectivity, by using ethanol (EtOH) as an environmentally benign electron donor and solvent.

## Results and discussion

### Synthesis and crystal structures

**Fig. 1:**
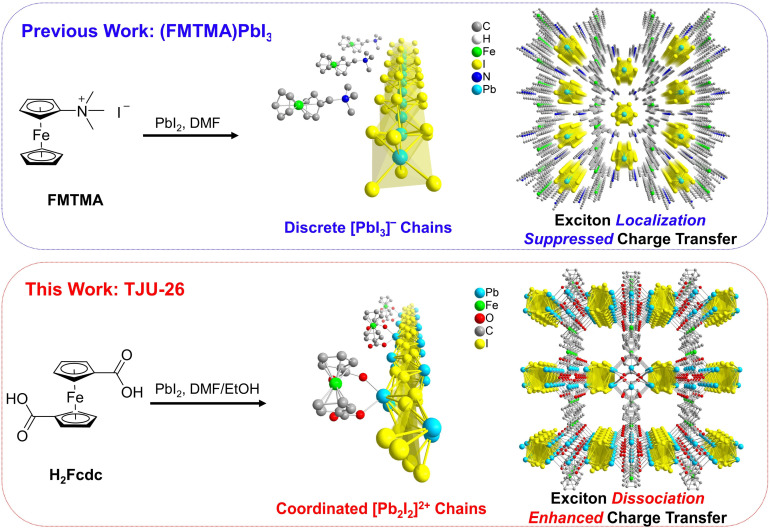
(Top) Synthetic scheme and crystallographic view of (FMTMA)PbI_3_, including a single [PbI_3_]^−^ chain with FMTMA^+^ cations and the overall structure of (FMTMA)PbI_3_. (Bottom) Synthetic scheme and crystallographic views of TJU-26, including a single [Pb_2_I_2_]^2+^ chain coordinated with Fcdc^2−^ ligands and the overall structure of TJU-26.

Conventional Fc-functionalized lead iodide perovskite, (FMTMA)PbI_3_ (FMTMA = ferrocenylmethyltrimethylammonium), was synthesized following a previously established procedure.^45^ This compound exhibits a typical ABX_3_-type perovskite structure, consisting of a hexagonal arrangement of infinite linear chains of face-sharing PbI_6_ octahedra along the *c* axis, with the FMTMA cations separating the chains through non-covalent electrostatic interactions (Fig. 1 top). In contrast, our ferrocene-functionalized lead iodide framework was synthesized by solvothermal reactions between PbI_2_ and 1,1′-ferrocenedicarboxylic acid (H_2_Fcdc) in DMF/EtOH, forming reddish-brown platelike crystals of [Pb_2_I_2_]^2+^[PbO][Fcdc^2−^], (TJU-26, TJU = Tongji University) (Fig. 1 bottom and S1†). X-ray crystallography reveals that the framework consists of an array of unidimensional linear [Pb_2_I_2_]^2+^ chains propagating along the *b*-axis (Fig. S2†). All of the iodide species are μ_3_-bridged among Pb^2+^ centers with Pb–I bond lengths between 3.287(1) and 3.590(1) Å. The iodo-plumbate chains are connected by square [Pb_2_O_2_] units, forming lead oxoiodide inorganic layers along the *ab* plane (Fig. S2†). Moreover, the Fcdc^2−^ ligands coordinatively decorate the Pb^2+^ centers to form a layered hybrid architecture (Fig. S3†). Moreover, the strong coordination bonding between the Pb^2+^ centers in the [Pb_2_I_2_]^2+^ chains and the carboxylate groups in the Fcdc^2−^ ligands is evidenced by the short Pb–O bond distances of 2.276–2.430 Å, which are significantly lower than the Pb–O distances of 2.753–2.872 Å observed between neutral [Pb_2_O_2_] units and Fcdc^2−^ ligands (Fig. S4†). Using carboxylate as the coordinating ligand, rather than conventional Fc-based organoammonium, facilitates an essential transition from an ionically bound lead halide perovskite to a lead halide framework, affording high structural stability and efficient charge-transfer behavior between the [Pb_2_I_2_]^2+^ chains and the Fcdc^2−^ ligands, as discussed in detail later.

**Fig. 2:**
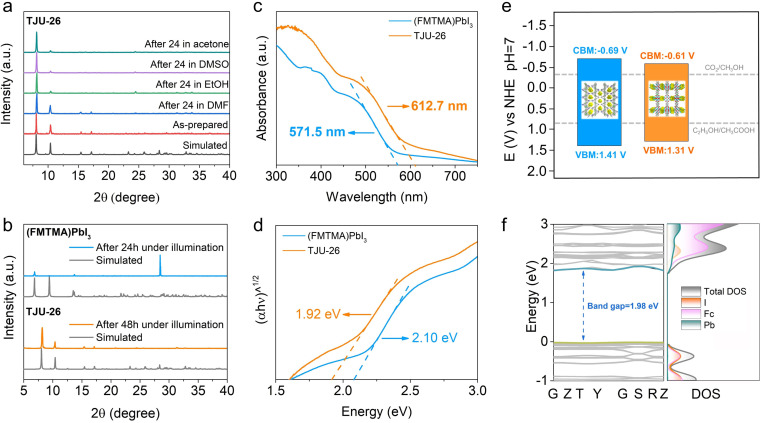
(a) PXRD patterns of TJU-26 before and after 24 h treatment in different organic solvents. (b) The PXRD patterns of TJU-26 and (FMTMA)PbI_3_ after light irradiation (300 W Xe lamp, AM 1.5G) for 48 h and 24 h, respectively. (c) Normalized UV-vis diffuse reflectance spectroscopy of TJU-26 and (FMTMA)PbI_3_. (d) Tauc plots of TJU-26 and (FMTMA)PbI_3_. (e) Band alignment of TJU-26 and (FMTMA)PbI_3_. (f) Calculated band structure and DOS of TJU-26.

A good match between experimental powder X-ray diffraction (PXRD) and the simulated pattern from the single-crystal data confirms the high yield and good phase purity of TJU-26 and (FMTMA)PbI_3_ (Fig. 2a and S5†). Fourier transform infrared (FT-IR) spectra suggest the presence and deprotonation of Fcdc^2−^ in TJU-26 (Fig. S6†). Stability measurements show that the as-synthesized (FMTMA)PbI_3_ crystals rapidly dissolve in polar organic solvents (*e.g.* DMF and DMSO). However, the as-synthesized TJU-26 is stable in these solvents for at least 24 h with no obvious loss in both crystallinity and mass balance, evidencing the high chemical stability of the coordination network (Fig. 2a). Thermogravimetric analysis (TGA) and *ex situ* thermodiffraction show high thermal stability up to 220 °C for TJU-26, higher than 200 °C for (FMTMA)PbI_3_ (Fig. S7 and S8†). Moreover, the photostability of TJU-26 was examined by continuous irradiation with AM 1.5G simulated sunlight (42 mW cm^−2^) for 48 h at room temperature. The overall stability of TJU-26 remained largely unaffected as confirmed by PXRD, while the slight discrepancy in absorption spectra is likely due to the partial surface modification during prolonged light irradiation (Fig. 2b and S9†). In contrast, a new diffraction peak (28.5°) in PXRD was clearly observed for (FMTMA)PbI_3_ after continuous irradiation for 24 h, suggesting the decomposition of the perovskite structure (Fig. 2b). Overall, the stability tests suggest that TJU-26 is a robust compound to perform photocatalysis in high-polarity protic solvents.

### Electronic band structure

Ultraviolet-visible (UV-vis) diffuse reflectance spectroscopy shows that TJU-26 has a broad visible absorption range with the absorption edge extending to 612.7 nm, superior to all of our previously reported lead halide frameworks (Fig. 2c).^33^ The band gap energy (*E*_g_) of TJU-26 was estimated to be 1.92 eV using the Tauc plot method (Fig. 2d). This enhancement in visible light absorption is attributed to the incorporation of the light-harvesting Fcdc^2−^ ligand, which substantially improves phonon absorption across the solar spectrum. The ferrocene-functionalized perovskite, (FMTMA)PbI_3_, exhibits a narrower visible absorption range with an absorption edge of 571.5 nm and a larger *E*_g_ of 2.10 eV (Fig. 2c and d). The reduced *E*_g_ of TJU-26 suggests that the formation of Pb^2+^-carboxylate coordination bonds between the Fcdc^2−^ and the lead iodide chains effectively enhances the conjugation of the framework.^47^ In addition, ultraviolet photoelectron spectroscopy (UPS) revealed that the valence band (VB) potentials (*vs.* the vacuum level) of TJU-26 and (FMTMA)PbI_3_ were −5.81 eV (+1.31 V *vs.* NHE pH 7) and −5.91 eV (+1.41 V *vs.* NHE pH 7), respectively (Fig. S10 and S11†). Their corresponding conduction band (CB) potentials (*vs.* the vacuum level) were −3.89 eV (−0.61 V *vs.* NHE pH 7) and −3.81 eV (−0.69 V *vs.* NHE pH 7), respectively. These values indicate that both TJU-26 and (FMTMA)PbI_3_ are thermodynamically capable of photocatalytic CO_2_ reduction coupled with EtOH oxidation (Fig. 2e).

DFT calculations suggest the calculated bandgap of TJU-26 to be 1.98 eV, close to the experimental value of 1.92 eV and again confirming its semiconductive nature (Fig. 2f). The total density of states (DOS) and the projected density of states (pDOS) on the Pb, I and Fcdc^2−^ ligands indicate that the valence band maximum (VBM) is dominated by I 5p orbitals, while Pb 6p orbitals largely contribute to the conduction band minimum (CBM) (Fig. 2f). The significant roles of Pb and I in frontier orbitals are analogous to the conventional hybrid lead halides, evidencing their contributions to carrier formation for photocatalysis.^48,49^ Meanwhile, owing to the Pb^2+^-carboxylate coordination motifs, the Fcdc^2−^ linkers are involved in frontier orbitals as well, which agrees with the potential ligand-to-metal charge transfer (Fig. 2f).^50^

### Charge transport dynamics

**Fig. 3:**
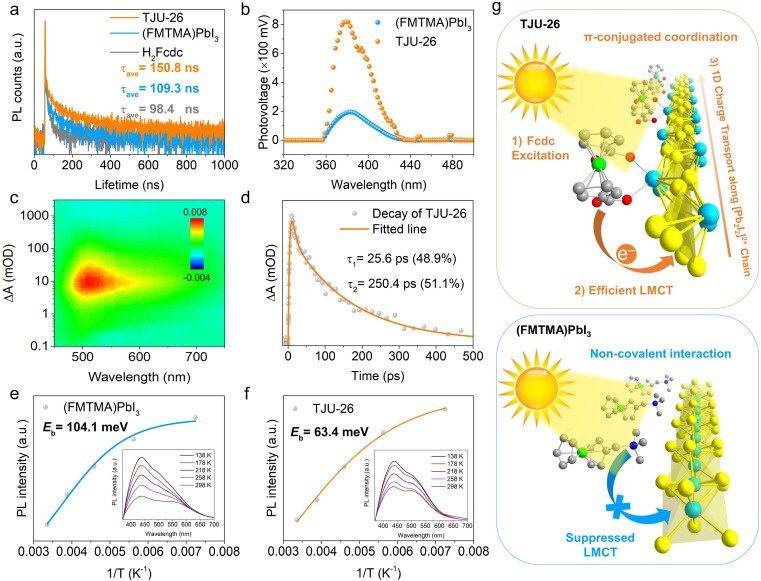
(a) Photoluminescence decay of H_2_Fcdc, (FMTMA)PbI_3_ and TJU-26 at room temperature. (b) SPV spectra of TJU-26 and (FMTMA)PbI_3_. (c) Two-dimensional pseudo-color TA plot of TJU-26. (d) TA kinetics of TJU-26. (e) Extracted exciton binding energies of TJU-26. (Inset) Temperature-dependent PL spectra of TJU-26 with an excitation wavelength of 355 nm. (f) Extracted exciton binding energies of (FMTMA)PbI_3_. (Inset) Temperature-dependent PL spectra of (FMTMA)PbI_3_ with an excitation wavelength of 350 nm. (g) Schematic presentation showing the photoexcitation, photoinduced LMCT and carrier transport of TJU-26 (left) and the suppressed photoinduced LMCT in (FMTMA)PbI_3_ (right).

Since the incorporation of the light-harvesting chromophore largely extended the visible-light absorption of TJU-26, it is important to investigate the LMCT process after the photoexcitation of the Fcdc^2−^ linkers and the e^−^–h^+^ separation. First, from the structural point of view, the π-conjugation between the cyclopentadiene ring and the carboxylate in the Fcdc^2−^ ligands allows for the transfer of delocalized electrons from Fc moieties to the soft [Pb_2_I_2_] chains, unlike ionic Fc-based perovskites (Fig. 1).^45^ Following this, the LMCT is evidenced by the substantial photoluminescence decrease of Fcdc^2−^ after incorporation into TJU-26, which exhibits a strong emission band at 379 nm for the free Fcdc^2−^ ligands (Fig. S12 and S13†). Meanwhile, TJU-26 shows largely quenched but broad photoluminescence ranging from 380 to 700 nm, with an average lifetime of 150.8 ns based on time-resolved photoluminescence (tr-PL) decay (Fig. 3a). The best fit of the decay provides a bi-exponential function with time constants of a long carrier lifetime of 601.7 ns and a short lifetime of 46.7 ns. Meanwhile, the tr-PL spectra of H_2_Fcdc and (FMTMA)PbI_3_ show that the average lifetimes are lower than that of TJU-26, with 98.4 ns for H_2_Fcdc and 109.3 ns for (FMTMA)PbI_3_, respectively (Fig. 3a). The higher PL lifetime of TJU-26 again supports the claim that the Pb^2+^-carboxylate coordination motifs between the conjugated Fcdc^2−^ ligand and the Pb^2+^ center are more favorable for the LMCT process. Indeed, DOS calculations show the contribution of the Fcdc^2−^ ligands and the Pb 6p orbitals to the VBM and CBM, respectively, agreeing with the claim of the LMCT process (Fig. 2f). In addition, the presence of the LMCT process in TJU-26 was confirmed by X-ray photoelectron spectroscopy (XPS). In the high-resolution XPS spectra of Fe 2p, the two peaks located at 710.6 eV and 723.1 eV are ascribed to Fe 2p_3/2_ and Fe 2p_1/2_, respectively.^51^ Meanwhile, a series of XPS characteristic peaks of Fe^3+^ (714.7 eV for Fe^3+^ 2p_3/2_ and 728.1 eV for Fe^3+^ 2p_1/2_) were also observed upon irradiation, which confirms the charge transfer process from the Fcdc^2−^ ligands to the Pb^2+^ sites (Fig. S14†).^52^

Importantly, LMCT is known to facilitate the separation and transport of photogenerated carriers, and surface photovoltage (SPV) spectroscopy is an important approach for investigating charge separation.^53^ The photovoltage (PV) onset of TJU-26 occurs at 2.86 eV (433 nm), and the maximum voltage of 819 mV is observed at 3.27 eV (379 nm) (Fig. 3b). Based on the band structure of TJU-26, the PV response is determined to be the charge separation of the band states. Given the SPV measurement of the TJU-26 film on FTO glass (see the Experimental section in the ESI†), the maximum theoretical photovoltage for the FTO/TJU-26 contact is 990 mV. Therefore, the experimental photovoltage of TJU-26 reaches ∼83% of this limit.^54,55^ However, the ionically bound Fc-based perovskite, (FMTMA)PbI_3_, exhibits a decreased maximum PV of 194 mV, corresponding to approximately 20% of the theoretical maximum PC (Fig. 3b). Despite the resemblance in SPV spectra, the nearly fourfold higher PV of TJU-26 indicates a significantly greater charge separation efficiency that is attributed to the efficient LMCT in TJU-26. Enhanced charge separation in TJU-26 is further supported by (photo)electrochemical studies. The photocurrent signal of TJU-26 is substantially higher than that of (FMTMA)PbI_3_, reflecting improved electron–hole separation efficiency (Fig. S16†).^56^ In addition, the smaller radius observed in the electrochemical impedance spectral (EIS) plots for TJU-26 implies a lower electron transfer resistance (Fig. S17†). The interfacial charge transfer resistance (*R*_ct_) for TJU-26 is 3.6 kΩ, significantly lower than the 24.9 kΩ measured for (FMTMA)PbI_3_, suggesting superior electron mobility in TJU-26.^57^

Hall effect measurements at room temperature reveal that TJU-26 exhibits n-type semiconductor behavior, with a carrier concentration of 9.8 × 10^13^ cm^−3^ and an estimated carrier mobility of ∼0.114 cm^2^ V^−1^ s^−1^. These values are comparable to those of many benchmark 3D lead halide perovskites and several orders of magnitude higher than those observed for 1D (FMTMA)PbI_3_ (4.1 × 10^10^ cm^−3^ and 0.032 cm^2^ V^−1^ s^−1^).^28,29^ This enhancement suggests that the incorporation of light-harvesting Fcdc^2−^ ligands improves visible-light photon absorption in TJU-26, followed by an LMCT process that further enhances charge separation. In addition, the average carrier diffusion lengths of TJU-26 and (FMTMA)PbI_3_ were determined to be 0.21 μm and 0.09 μm, respectively, based on the equation1
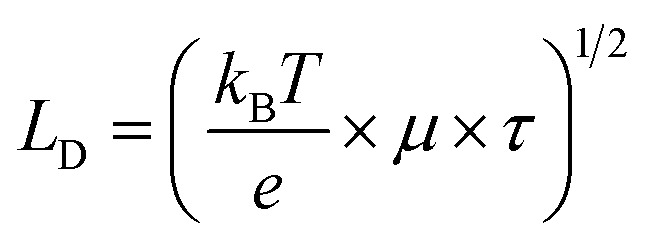
where *k*_B_ is Boltzmann's constant, *T* is the temperature, *e* is the electron charge, *μ* is the Hall mobility, and *τ* is the carrier lifetime. Moreover, the effective masses of charge carriers in TJU-26 were calculated to be 2.10 *m*_0_ for electrons 
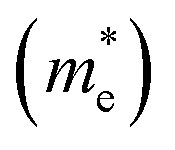
 and 0.51 *m*_0_ for holes 
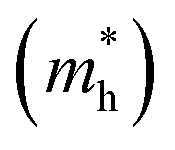
, respectively. It is well established that small carrier effective masses contribute to high carrier mobility and long carrier diffusion lengths.^58^

To obtain detailed insights into the charge carrier dynamics of TJU-26 under light irradiation, femtosecond transient absorption (fs-TA) spectroscopy was performed upon 320 nm excitation. The pseudo-color fs-TA spectra of TJU-26 exhibit a positive signal across the 450–800 nm range, with a maximum at 515 nm (Fig. 3c). This signal is attributed to the absorption from photoinduced electron transfer and directly reveals the process of charge separation within 1D lead iodide chains under light exposure.^59^ This signal reaches its maximum value at ∼10 ps at 515 nm, suggesting a high rate of charge separation (Fig. S18†). However, the peak at 515 nm rapidly weakens around 50 ps, with a concomitant appearance of a new peak at 667 nm, indicating a change in the interactions between photogenerated electrons and holes (Fig. S18†).^60^ This new observation is attributed to electron transfer from the Fcdc^2−^ ligand to the [Pb_2_I_2_]^2+^ SBUs *via* the Pb^2+^-carboxylate linkage under photoexcitation, which facilitates spatial charge separation and transport. Analysis of the recovery kinetics at 515 nm in the TA spectra shows that the decay of TJU-26 follows a biexponential function with two time constants, *τ*_1_ = 25.6 ps and *τ*_2_ = 250.4 ps (Fig. 3d), resulting in a long average lifetime of 122.6 ps. These findings indicate that the charge transfer between the Fcdc^2−^ ligand and the lead halide structural unit probably delays TA decay kinetics, thereby suppressing electron–hole recombination.^61^

To further investigate the exciton dissociation behaviors of TJU-26 and (FMTMA)PbI_3_, temperature-dependent photoluminescence (PL) measurements were conducted to determine their exciton binding energies (*E*_b_) (Fig. 3e and f). The PL intensities of both materials decreased as the temperature increased, primarily due to thermally activated nonradiative recombination processes.^62^ The *E*_b_ was estimated by combining integrated PL intensity with temperature, using the following equation:2
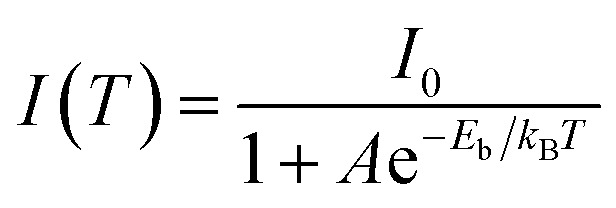
where *I*_0_ is the PL intensity at 0 K, *A* is a proportional constant, and *k*_B_ is the Boltzmann constant. By fitting the experimental data, the *E*_b_ values of TJU-26 and (FMTMA)PbI_3_ were determined to be 63.4 meV and 104.1 meV, respectively (Fig. 3e and f). TJU-26 exhibited a lower *E*_b_ than (FMTMA)PbI_3_, suggesting the high tendency of electrons and hole dissociation into free carriers in TJU-26.^63^ These photophysical studies indicate that the coordination linkage between Fcdc^2−^ and Pb^2+^ centers facilitates an effective LMCT process, which not only enhances carrier transport but also promotes exciton dissociation within the [Pb_2_I_2_]^2+^ chains, making TJU-26 a promising candidate for multi-e^−^ CO_2_ photoreduction (Fig. 3g).

### Photocatalytic CO_2_-to-CH_3_OH conversion

**Fig. 4:**
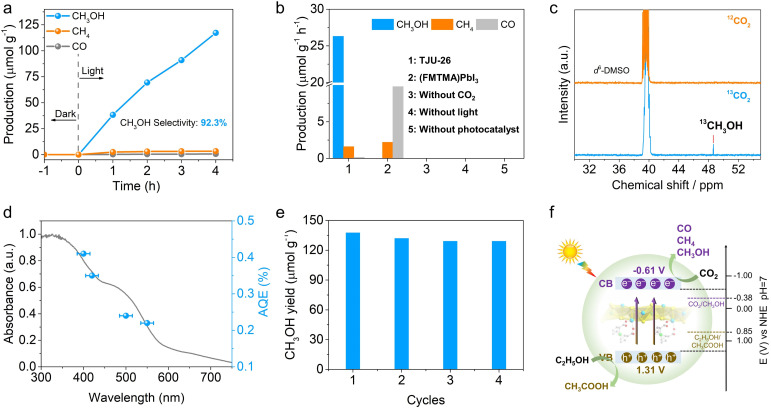
(a) Time courses of CH_3_OH, CH_4_ and CO evolution for photocatalytic CO_2_ reduction by TJU-26 in EtOH (AM1.5G simulated sunlight). (b) The control experiments of photocatalytic CO_2_ reduction performance over TJU-26 and (FMTMA)PbI_3_. (c) ^13^C NMR results of ^13^CH_3_OH produced over TJU-26 from the ^13^CO_2_ isotope experiment. (d) Wavelength-dependent AQEs of photocatalytic CO_2_ reduction on TJU-26. Error bars represent the deviations of monochromatic light wavelengths. (e) Time courses of CH_3_OH production over 20 mg of TJU-26 in 10 mL of EtOH for four consecutive cycles. (f) Schematic diagram for photocatalytic CO_2_ reduction by TJU-26.

Given the suitable band positions and excellent carrier separation/transport of TJU-26, CO_2_ photoreduction was studied by introducing 20 mg as-synthesized TJU-26 into a sealed reaction system containing 10 mL EtOH and 1 atm CO_2_. No additional photosensitizer, metal co-catalyst or sacrificial reducing agent was necessary for this photocatalytic system. EtOH functions not only as a donor scavenger, accepting photogenerated holes, but also as an environmentally benign solvent. Both ^1^H nuclear magnetic resonance (NMR) spectroscopy and gas chromatography (GC) have been employed to quantitatively identify the reduction products. Upon AM 1.5G simulated illumination, TJU-26 steadily catalyzed CO_2_ reduction to produce CH_3_OH as the major product over a time span of 4 h (Fig. 4a and S19†). A linear increment in the CH_3_OH yields was observed and the average CH_3_OH evolution rate was determined to be 26.31 μmol h^−1^ g^−1^, while trace amounts of CO (0.14 μmol h^−1^ g^−1^) and CH_4_ (1.60 μmol h^−1^ g^−1^) were detected as well (Fig. 4a). These values correspond to photocatalytic selectivities of 92.3% (electron basis) and 93.7% (product basis) for CO_2_-to-CH_3_OH transformation, which are among the highest reported values for hybrid lead halides (Table S2†). Moreover, the CO_2_ photoreduction performance of TJU-26 is comparable to that of benchmark CO_2_-to-CH_3_OH photocatalysts, such as carbon dot-based photocatalysts (^m^CD) (13.9 μmol h^−1^ g^−1^) and Bi_19_Br_3_S_27_ (0.62 μmol h^−1^ g^−1^) (Table S3†).^7,16^

Control experiments conducted in the absence of TJU-26, light, or CO_2_ yielded no methanol, confirming that the methanol formation results from the CO_2_ reduction reaction catalyzed by TJU-26 (Fig. 4b). To further confirm the origin of the carbon source, a control photocatalytic experiment was carried out using ^13^C isotopically labelled CO_2_. The chemical shift signal peak at 48.6 ppm (^13^CH_3_OH) was clearly observed by ^13^C NMR spectroscopy. Meanwhile, no peak was observed at 48.6 ppm using ^12^CO_2_ under identical reaction conditions, demonstrating that the CH_3_OH product arises from the reduction of CO_2_ instead of other carbon sources (Fig. 4c). In order to confirm the multi-electron CO_2_ reduction process, the kinetically favorable 2e^−^ reduced product (*i.e.* formic acid, HCOOH) is employed as the precursor. Photocatalysis in a closed reaction system containing 40 mg TJU-26 and 20 μL formic acid in 8 mL EtOH without CO_2_ produces CH_3_OH as the major product at an evolution rate of 29.54 μmol h^−1^ g^−1^ (Fig. S20†). This set of experiments confirm that the populated carrier accumulation nature of TJU-26 enables the further reduction of 2e^−^ intermediates to afford the final product of CH_3_OH. Moreover, we have performed control photocatalysis experiments to exclude the presence of ethanol, using sodium sulfite as the hole scavenger in aqueous solution instead. In this system, the evolution rates of CH_3_OH, CH_4_, and CO were measured to be 10.53 μmol h^−1^ g^−1^, 0.67 μmol h^−1^ g^−1^, and 6.34 μmol h^−1^ g^−1^, respectively (Fig. S21†). These results provide further evidence that methanol is derived from the photoreduction of CO_2_, rather than from the decomposition of ethanol.

The wavelength-dependent apparent quantum efficiencies (AQEs) were measured to be 0.41% at 400 nm (±15 nm), 0.35% at 420 nm (±15 nm), 0.24% at 500 nm (±15 nm) and 0.22% at 550 nm (±15 nm) (Fig. 4d). The trend is consistent with the UV-vis absorption of TJU-26, implying an intrinsic photo-driven catalytic process. In control experiments, (FMTMA)PbI_3_ was measured under identical photocatalytic conditions, resulting in CO_2_ reduction products of CO and CH_4_ with evolution rates of 9.83 μmol h^−1^ g^−1^ and 2.21 μmol h^−1^ g^−1^, respectively (Fig. 4b). The significantly enhanced photocatalytic CO_2_ reduction performance of TJU-26 again confirms the essential role of the π-conjugated coordination linkage between Fcdc^2−^ ligands and [Pb_2_I_2_]^2+^ chains in facilitating charge separation and transport (Fig. 3g).

The long-term photocatalytic stability of TJU-26 was studied by performing photocatalysis under continuous AM 1.5G illumination for a total of 16 h, with evacuation and CO_2_ refilling every 4 h (Fig. 4e). The photocatalyst retained over 93% of the initial CO_2_ photoreduction activity for the next three runs, showing the excellent durability of TJU-26 under the photocatalytic conditions. Moreover, PXRD, UV-vis and FTIR spectroscopy synergistically indicate that TJU-26 largely retains its structural integrity after photocatalysis (Fig. S22–S24†). Indeed, a negligible amount of Pb^2+^ leaching was noticed in the solution after the photocatalytic reaction (1.12 ppm by inductively-coupled plasma optical emission spectroscopy, ICP-OES). Meanwhile, the role of EtOH as an electron donor was confirmed by the presence of acetic acid (CH_3_COOH) as the oxidized product by ^1^H NMR, which was performed by a continuous photocatalytic reaction for 12 h (Fig. 4f). The quantitative characterization of acetic acid was performed by using DMSO as the internal standard, corresponding to an evolution rate of 32.2 μmol h^−1^ g^−1^ (Fig. S25†). This value accounts for ∼83% of the theoretical yield for the oxidation product, demonstrating that CH_3_CH_2_OH-to-CH_3_COOH oxidation is the major coupling reaction in CO_2_ photoreduction. Notably, coupling CO_2_ photoreduction with alcohol oxidation has emerged as an appealing approach for synergistic utilization of photogenerated electrons and holes, offering an advantage over the conventional use of hole scavengers such as triethylamine (TEA).^64^

### Photocatalytic mechanism

**Fig. 5:**
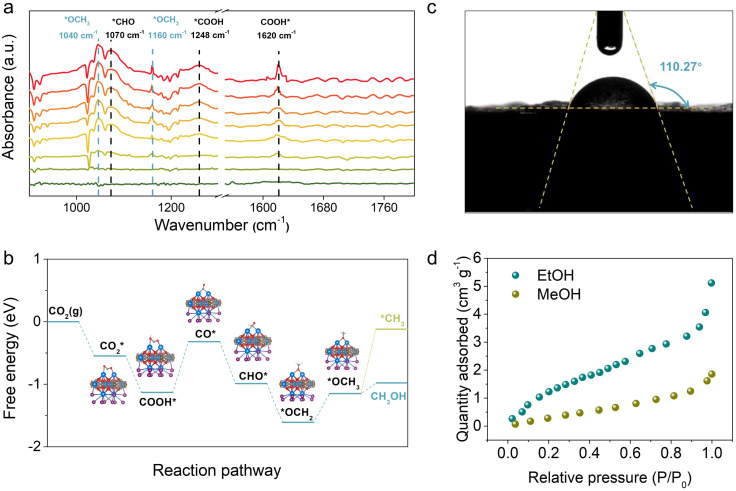
(a) *In situ* DRIFTS spectra for the photocatalytic CO_2_ reduction process by TJU-26. (b) Free energy diagram of photocatalytic CO_2_-to-CH_3_OH transformation on the (001) facet of TJU-26. (c) Contact angle measurement of TJU-26 with water. (d) Vapor adsorption isotherms of TJU-26 at 298 K.

To understand the photoreaction mechanism on TJU-26, the surface species evolved in the reaction were monitored by *in situ* diffuse reflectance infrared Fourier transform spectroscopy (DRIFTS) (Fig. 5a). First, during the DRIFT studies, no intermediate was observed in N_2_ or in the dark under identical conditions, suggesting the CO_2_ photoreduction process. When performing CO_2_ catalysis with light irradiation, a prominent band at 1620 cm^−1^ for carboxyl stretching is gradually observed with the irradiation time increasing from 0 to 60 min. This peak suggests the presence of *COOH species as one of the intermediates.^65,66^ The *CHO intermediate with a characteristic band at 1070 cm^−1^ is noticed as well, implying the possible protonation of COOH* to afford *CO and the follow-up proton transfer to form *CHO.^67^ More importantly, the concomitant absorption bands at 1040 cm^−1^ and 1160 cm^−1^ are clearly observed, suggesting the presence of H_3_CO*, which is one of the key intermediates during the CO_2_-to-CH_3_OH transformation.^7,68^ According to the above results, it is speculated that the most likely reduction pathway could be as follows:3
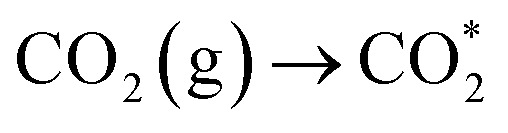
4
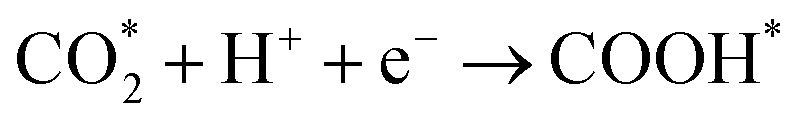

5 COOH* + H^+^ + e^−^ → *CO + H_2_O

6 *CO + H^+^ + e^−^ → *CHO

7 *CHO + H^+^ + e^−^ → H_2_CO*

8 H_2_CO* + H^+^ + e^−^ → H_3_CO*

9 H_3_CO* + H^+^ + e^−^ → CH_3_OH

where “*” represents the corresponding adsorption sites on the surface of the photocatalyst.

Following this, we have explored the active sites for photocatalytic CO_2_ reduction on TJU-26 by quasi *in situ* XPS spectroscopy. Under light irradiation, the I 3d peak shifted to higher binding energy, indicating a decrease in the electron density around the I site (Fig. S26†).^69^ Meanwhile, we have observed characteristic peaks of both Fe^2+^ and Fe^3+^ in the high-resolution Fe 2p spectra, and the percentage of Fe^3+^ increased substantially under light irradiation, which was attributed to the charge transfer from the Fcdc^2−^ ligands to the Pb^2+^ sites through the Pb^2+^-carboxylate coordination bridge (Fig. S14 and S15†).^52^ Indeed, the Pb 4f peak shifted to lower binding energy, indicating that the electron density around the Pb^2+^ site in TJU-26 increased after light irradiation (Fig. S27†).^70^ Overall, the increase in the electron density of the Pb^2+^ sites in TJU-26 facilitates CO_2_ adsorption and activation.

To further understand the reaction mechanism, we have performed Gibbs free energy calculations using the (001) facet as the representative surface of TJU-26 to model the possible reaction pathways involved in the CO_2_-to-CH_3_OH process. First, the CO_2_ adsorption calculations indicate that the CO_2_ adsorption and activation on TJU-26 are thermodynamically favorable (Fig. 5b). Importantly, the formation of the COOH* intermediate (eqn (4)) is found to be a spontaneous process on TJU-26, which is often the rate-determining step in many previous studies.^7^ In this exothermic step, the oxygens of the COOH* species preferentially bind with two adjacent Pb^2+^ sites in a bidentate manner, resulting from the suitable Pb⋯Pb distance of 3.691 Å in the lead oxyiodide layers. To note, both Pb^2+^ centers originate from the [Pb_2_I_2_]^2+^ chains instead of [Pb_2_O_2_] clusters, verifying that the iodoplumbate species indeed serve as photocatalytically active sites. The protonation of *COOH to produce *CO by TJU-26 has a low free-energy barrier of 0.81 eV to form the C-anchored *CO intermediate. This reaction pathway is further confirmed by a control experiment using formic acid instead of CO_2_ as the precursor to produce CH_3_OH at an evolution rate of 29.54 μmol h^−1^ g^−1^. Since the carbon of *CO has a contact distance of 2.175 Å with one Pb^2+^ site, the moderate adsorption strength of *CO tends to accept a proton–electron pair to form *CHO (eqn (6)) instead of CO desorption as the by-product (evolution rate of 0.14 μmol g^−1^ h^−1^). The thermodynamically favorable process for the conversion of *CO to *CHO is supported by both DFT calculations and the observed *CHO species (1070 cm^−1^) in the aforementioned *in situ* DRIFT studies (Fig. 5a). Following this, DFT calculations suggest that the reaction undergoes the formyl pathway, where the addition of another electron–hole pair leads to the formation of H_2_CO* through an exothermic process (eqn (7)), thanks to the bidentate binding motif with dual Pb^2+^ centers. This suggests the important transition from C-anchored *CHO to O-anchored H_2_CO* on TJU-26 (Fig. 5b). Subsequently, H_2_CO* accepts one more electron–hole pair to form CH_3_O*, which agrees with the *in situ* DRIFT observations. Although the hydrogenation of *OCH_3_ to CH_3_OH is an uphill process, the free-energy change for this step is much smaller than that for the conversion of *OCH_3_ to *CH_3_*via* the acceptance of two e^−^/H^+^ pairs (Fig. 5b). This effectively rules out the possibility of CH_4_ being the major product on TJU-26 (Fig. 5b). Overall, the reaction mechanism for the photocatalytic conversion of CO_2_ to CH_3_OH has been thoroughly investigated through a combination of *in situ* spectroscopic experiments and theoretical calculations.

Since the major oxidation half-reaction is experimentally determined to be EtOH oxidation, the competitive absorption behaviors of TJU-26 towards CH_3_OH and EtOH are studied using vapor adsorption isotherms and contact angle measurements. First, the hydrophobic nature of TJU-26 is confirmed by an average contact angle of 110.27 , suggesting that TJU-26 has a higher affinity towards EtOH rather than CH_3_OH (Fig. 5c). The vapor adsorption isotherms of TJU-26 indicate the uptake amounts of 5.12 cm^3^ g^−1^ for EtOH and 1.86 cm^3^ g^−1^ for CH_3_OH at 298 K under 1 bar (Fig. 5d). The substantially higher amount of EtOH adsorption suggests strong interactions between EtOH and TJU-26. Since the layered material has no apparent open pore window, the vapor adsorption probably occurs on the crystal surfaces of TJU-26. Given the substantially higher EtOH adsorption over CH_3_OH adsorption, TJU-26 favors the desorption of CH_3_OH as the product that agrees with the low desorption barrier of 0.17 eV based on DFT calculations (Fig. 5b).

## Conclusions

In this study, we present the first example of a hybrid lead halide that can achieve photocatalytic CO_2_-to-CH_3_OH transformation. By incorporating an Fc-based ligand as a light-harvesting antenna into the ultrastable lead iodide framework, its visible-light absorption edge is extended to over 600 nm, followed by an efficient LMCT process that triggers carrier transport along the linear lead iodide chains. Compared to the control ionic Fc-based perovskite ((FMTMA)PbI_3_), our lead iodide framework exhibits synergistic interactions between inorganic and organic photoactive components for enhanced carrier transport by multiple orders of magnitude. Notably, TJU-26 demonstrates high structural robustness in high-polar protic environments and achieves photocatalytic CO_2_ reduction to high value-added CH_3_OH under AM 1.5G irradiation, whereas most hybrid metal halides produce CO as the primary product. The AQE of TJU-26 for CH_3_OH production reaches 0.41% at 400 nm, comparable to those of many benchmark photocatalysts (*e.g.*, ^m^CD/CN). Importantly, the oxidative half-reaction coupled with CO_2_ photoreduction involves environmentally benign EtOH oxidation, resulting in the overall photocatalysis reaction in a sustainable manner. *In situ* DRIFT experiments and DFT calculations clearly indicate that the photocatalysis undergoes the formyl pathway to afford the key intermediate CH_3_O*, which occurs on the dual Pb_2_ active sites residing in [Pb_2_I_2_]^2+^ chains. Given the numerous examples of light-harvesting antenna-functionalized organocarboxylate linkers, we believe that this study opens up many possibilities for chemical functionalization of ultrastable lead halide frameworks for a variety of photoactive applications.

## Data availability

The data that support the findings of this study are available from the corresponding author upon reasonable request.

## Author contributions

J. Y., Y. H. and H. F. conceived the project. H. F. supervised the project. J. Y. and Y. H. performed the majority of experimental studies. C. S. assisted in the crystallographic analysis. Y. J. assisted in the synthesis and photophysical studies. J. Y., Y. H. and H. F. wrote and revised the manuscript. All authors contributed to discussion and revisions.

## Conflicts of interest

There are no conflicts to declare.

## Supplementary Material

SC-OLF-D4SC08216H-s001

SC-OLF-D4SC08216H-s002

## References

[cit1] Graciani J., Mudiyanselage K., Xu F., Baber A. E., Evans J., Senanayake S. D., Stacchiola D. J., Liu P., Hrbek J., Sanz J. F., Rodriguez J. A. (2014). Science.

[cit2] Kar S., Sen R., Goeppert A., Prakash G. K. S. (2018). J. Am. Chem. Soc..

[cit3] Liu C., Yang B., Tyo E., Seifert S., DeBartolo J., von Issendorff B., Zapol P., Vajda S., Curtiss L. A. (2015). J. Am. Chem. Soc..

[cit4] Habisreutinger S. N., Schmidt-Mende L., Stolarczyk J. K. (2013). Angew. Chem., Int. Ed..

[cit5] Navarro-Jaén S., Virginie M., Bonin J., Robert M., Wojcieszak R., Khodakov A. Y. (2021). Nat. Rev. Chem.

[cit6] Roy S. C., Varghese O. K., Paulose M., Grimes C. A. (2010). ACS Nano.

[cit7] Li J., Pan W., Liu Q., Chen Z., Chen Z., Feng X., Chen H. (2021). J. Am. Chem. Soc..

[cit8] Lu K.-Q., Li Y.-H., Zhang F., Qi M.-Y., Chen X., Tang Z.-R., Yamada Y. M. A., Anpo M., Conte M., Xu Y.-J. (2020). Nat. Commun..

[cit9] Li Y., Li B., Zhang D., Cheng L., Xiang Q. (2020). ACS Nano.

[cit10] Jiang Z., Sun H., Wang T., Wang B., Wei W., Li H., Yuan S., An T., Zhao H., Yu J., Wong P. K. (2018). Energy Environ. Sci..

[cit11] Liu D., Chen D., Li N., Xu Q., Li H., He J., Lu J. (2020). Angew. Chem., Int. Ed..

[cit12] Gong E., Ali S., Hiragond C. B., Kim H. S., Powar N. S., Kim D., Kim H., In S.-I. (2022). Energy Environ. Sci..

[cit13] Olah G. A. (2005). Angew. Chem., Int. Ed..

[cit14] Natte K., Neumann H., Beller M., Jagadeesh R. V. (2017). Angew. Chem., Int. Ed..

[cit15] Goeppert A., Czaun M., Jones J.-P., Surya Prakash G. K., Olah G. A. (2014). Chem. Soc. Rev..

[cit16] Wang Y., Liu X., Han X., Godin R., Chen J., Zhou W., Jiang C., Thompson J. F., Mustafa K. B., Shevlin S. A., Durrant J. R., Guo Z., Tang J. (2020). Nat. Commun..

[cit17] Tu W., Zhou Y., Zou Z. (2014). Adv. Mater..

[cit18] Lais A., Gondal M. A., Dastageer M. A. (2018). Environ. Chem. Lett..

[cit19] Park S., Chang W. J., Lee C. W., Park S., Ahn H.-Y., Nam K. T. (2016). Nat. Energy.

[cit20] Wu Y., Wang P., Zhu X., Zhang Q., Wang Z., Liu Y., Zou G., Dai Y., Whangbo M.-H., Huang B. (2018). Adv. Mater..

[cit21] Wu Y., Wang P., Guan Z., Liu J., Wang Z., Zheng Z., Jin S., Dai Y., Whangbo M.-H., Huang B. (2018). ACS Catal..

[cit22] Xu Y.-F., Yang M.-Z., Chen B.-X., Wang X.-D., Chen H.-Y., Kuang D.-B., Su C.-Y. (2017). J. Am. Chem. Soc..

[cit23] Hong Z., Chong W. K., Ng A. Y. R., Li M., Ganguly R., Sum T. C., Soo H. S. (2019). Angew. Chem., Int. Ed..

[cit24] Tang C., Chen C., Xu W., Xu L. (2019). J. Mater. Chem. A.

[cit25] Zhu X., Lin Y., Sun Y., Beard M. C., Yan Y. (2019). J. Am. Chem. Soc..

[cit26] Chen K., Deng X., Dodekatos G., Tüysüz H. (2017). J. Am. Chem. Soc..

[cit27] Brenner T. M., Egger D. A., Kronik L., Hodes G., Cahen D. (2016). Nat. Rev. Mater..

[cit28] Shi D., Adinolfi V., Comin R., Yuan M., Alarousu E., Buin A., Chen Y., Hoogland S., Rothenberger A., Katsiev K., Losovyj Y., Zhang X., Dowben P. A., Mohammed O. F., Sargent E. H., Bakr O. M. (2015). Science.

[cit29] Xing G., Mathews N., Sun S., Lim S. S., Lam Y. M., Grätzel M., Mhaisalkar S., Sum T. C. (2013). Science.

[cit30] Jeon N. J., Noh J. H., Kim Y. C., Yang W. S., Ryu S., Seok S. I. (2014). Nat. Mater..

[cit31] Jung M., Ji S.-G., Kim G., Seok S. I. (2019). Chem. Soc. Rev..

[cit32] Huang H., Pradhan B., Hofkens J., Roeffaers M. B. J., Steele J. A. (2020). ACS Energy Lett..

[cit33] Sun C., Xi R., Fei H. (2023). Acc. Chem. Res..

[cit34] Zhuang Z., Peng C., Zhang G., Yang H., Yin J., Fei H. (2017). Angew. Chem., Int. Ed..

[cit35] Yin J., Yang H., Fei H. (2019). Chem. Mater..

[cit36] Peng C., Song X., Yin J., Zhang G., Fei H. (2019). Angew. Chem., Int. Ed..

[cit37] Yin J., Yu Y., Song X., Jiang Y., Fei H. (2021). CCS Chem..

[cit38] Peng C., Zhuang Z., Yang H., Zhang G., Fei H. (2018). Chem. Sci..

[cit39] Jiang Y., Yin J., Xi R., Fei H. (2024). Chem. Sci..

[cit40] Song X., Wei G., Sun J., Peng C., Yin J., Zhang X., Jiang Y., Fei H. (2020). Nat. Catal..

[cit41] Chen X., Peng C., Dan W., Yu L., Wu Y., Fei H. (2022). Nat. Commun..

[cit42] Singh S. K., Chauhan R., Singh B., Diwan K., Kociok-Köhn G., Bahadur L., Singh N. (2012). Dalton Trans..

[cit43] Yao S.-J., Li N., Liu J., Dong L.-Z., Liu J.-J., Xin Z.-F., Li D.-S., Li S.-L., Lan Y.-Q. (2022). Inorg. Chem..

[cit44] Fillafer N., Kuper H., Schaate A., Locmelis S., Becker J. A., Krysiak Y., Polarz S. (2022). Adv. Funct. Mater..

[cit45] Zhang Z.-X., Zhang H.-Y., Zhang W., Chen X.-G., Wang H., Xiong R.-G. (2020). J. Am. Chem. Soc..

[cit46] Du C., Sheng J., Zhong F., He Y., Liu H., Sun Y., Dong F. (2024). Proc. Natl. Acad. Sci. U. S. A..

[cit47] Sun H., Zhao Z., Xing Y., Wei K., Zhao X., Zhao Y., Wang X., Kang Z., Li Y., Tan H. (2024). Adv. Funct. Mater..

[cit48] Zhou C., Lin H., Shi H., Tian Y., Pak C., Shatruk M., Zhou Y., Djurovich P., Du M.-H., Ma B. (2018). Angew. Chem., Int. Ed..

[cit49] Smith M. D., Connor B. A., Karunadasa H. I. (2019). Chem. Rev..

[cit50] Li S., Li Z., Yue J., Wang H., Wang Y., Su W., Waterhouse G. I. N., Liu L., Zhang W., Zhao Y. (2024). Angew. Chem., Int. Ed..

[cit51] Zhang H., Si S., Zhai G., Li Y., Liu Y., Cheng H., Wang Z., Wang P., Zheng Z., Dai Y., Liu T. X., Huang B. (2023). Appl. Catal., B.

[cit52] Xia L., Zhou W., Xu Y., Xia Z., Wang X., Yang Q., Xie G., Chen S., Gao S. (2023). Chem.
Eng. J..

[cit53] Wu X.-P., Gagliardi L., Truhlar D. G. (2018). J. Am. Chem. Soc..

[cit54] Melo Jr M. A., Wu Z., Nail B. A., De Denko A. T., Nogueira A. F., Osterloh F. E. (2018). Nano Lett..

[cit55] Wang J., Zhao J., Osterloh F. E. (2015). Energy Environ. Sci..

[cit56] Shi Y., Zhan G., Li H., Wang X., Liu X., Shi L., Wei K., Ling C., Li Z., Wang H., Mao C., Liu X., Zhang L. (2021). Adv. Mater..

[cit57] Zhang H., Liu S., Zheng A., Wang P., Zheng Z., Wang Z., Cheng H., Dai Y., Huang B., Liu Y. (2024). Angew. Chem., Int. Ed..

[cit58] Wang X., Li T., Xing B., Faizan M., Biswas K., Zhang L. (2021). J. Phys. Chem. Lett..

[cit59] Li S., Wei W., Chi K., Ferguson C. T. J., Zhao Y., Zhang K. A. I. (2024). J. Am. Chem. Soc..

[cit60] Cheng Y.-Z., Ji W., Wu X., Ding X., Liu X.-F., Han B.-H. (2022). Appl. Catal., B.

[cit61] Liu Z., Yin H., Sun J., Bai L., Li Z., Zhao X., Yan X., Zhao M., Jing L. (2024). Adv. Energy Mater..

[cit62] Fu G., Yang D., Xu S., Li S., Zhao Y., Yang H., Wu D., Petkov P. S., Lan Z.-A., Wang X., Zhang T. (2024). J. Am. Chem. Soc..

[cit63] Cheng C., Zhang S., Zhang J., Guan L., El-Khouly M. E., Jin S. (2024). Angew. Chem., Int. Ed..

[cit64] Zhang W., Song C.-C., Wang J.-W., Cai S.-T., Gao M.-Y., Feng Y.-X., Lu T.-B. (2023). Chin. J. Catal..

[cit65] Li X., Sun Y., Xu J., Shao Y., Wu J., Xu X., Pan Y., Ju H., Zhu J., Xie Y. (2019). Nat. Energy.

[cit66] Chen P., Lei B., Dong X. a., Wang H., Sheng J., Cui W., Li J., Sun Y., Wang Z., Dong F. (2020). ACS Nano.

[cit67] Xu J., Ju Z., Zhang W., Pan Y., Zhu J., Mao J., Zheng X., Fu H., Yuan M., Chen H., Li R. (2021). Angew. Chem., Int. Ed..

[cit68] Wang Y., Chen E., Tang J. (2022). ACS Catal..

[cit69] Ban C., Wang Y., Feng Y., Zhu Z., Duan Y., Ma J., Zhang X., Liu X., Zhou K., Zou H., Yu D., Tao X., Gan L., Han G., Zhou X. (2024). Energy Environ. Sci..

[cit70] Zhao L., Bian J., Zhang X., Bai L., Xu L., Qu Y., Li Z., Li Y., Jing L. (2022). Adv. Mater..

