# Supplementary material for: Coordination-driven assembly of a ferrocene-functionalized lead iodide framework with enhanced stability and charge transfer for photocatalytic CO2-to-CH3OH conversion
Source: Chem Sci. 2025 Mar 31;16(19):8327–37. doi: 10.1039/d4sc08216h (PMC11979706; doi:10.1039/d4sc08216h)
Supplement: SC-016-D4SC08216H-s001 [file SC-016-D4SC08216H-s001.pdf]

Supplementary Information (SI) for Chemical Science.
This journal is © The Royal Society of Chemistry 2025

Supporting Information

Coordination-Driven Assembly of a Ferrocene-Functionalized Lead Iodide Framework with Enhanced Stability and Charge Transfer for Photocatalytic CO_2-to-CH_3OH Conversion

*Jinlin Yin,[†] Yani He,[†] Chen Sun, Yilin Jiang, and Honghan Fei**

Shanghai Key Laboratory of Chemical Assessment and Sustainability, School of Chemical Science and Engineering, Tongji University, 1239 Siping Rd., Shanghai 200092 (China)

Corresponding Author

E-mail: fei@tongji.edu.cn

Author Contributions

[†] These authors contributed equally.

Experimental Section

Synthesis of (FMTMA)PbI₃. A modified approach based on a previous publication was used to synthesize high-quality (FMTMA)PbI_3.[S1] Specifically, PbI_2 (1.15 g, 2.5 mmol) were dissolved in DMF (100 mL) with stirring. Equimolar (ferrocenylmethyl)trimethylammonium iodide was then added to the solution while stirring and heating. Dark-brown crystals of (FMTMA)PbI_3 were obtained via slow evaporation at 333 K.

Synthesis of TJU-26. In a typical solvothermal synthesis, a mixture of PbI_2 (0.46 g, 1 mmol), 1,1'-ferrocenedicarboxylic acid (H_2Fcdc, 0.27 g, 1 mmol) and 12 mL mixed solvent of ethanol and DMF ($V_{EtOH} : V_{DMF} = 2 : 1$) were added into a 20 mL Teflon-lined autoclave reactor, followed by 30 min of vigorous stirring for a sufficient dispersion. The autoclave was then sealed into a stainless-steel vessel and heated statically at 140 °C for 24 h. After cooling down to room temperature, the solids were separated by vacuum filtration, rinsed extensively with anhydrous ethanol, and then dried at 60 °C for 6 h to afford reddish-brown crystals of TJU-26 with a yield of 75% (0.29 g, based on Pb). Element analysis: calculated C, 13.89%; H, 0.77%; found C, 13.11%; H, 0.78%.

Single crystal X-ray crystallography. A suitable single crystal of TJU-26 was selected under an optical microscope, and mounted onto a glass fiber. The diffraction data of the pristine single crystal were collected at ambient temperature using graphite-monochromated Mo-Kα radiation (λ =0.71073 Å), operated at 50 kV and 30 mA on a Bruker SMART APEX II CCD area detector X-ray diffractometer. The diffraction scans method using a combination of phi and omega scans with the scan speeds of 3 s/° for the phi scans and 1 s/° for the omega scans at 2θ = 0°. The crystal structure was solved by direct methods and expanded routinely. The model was refined by fullmatrix least-squares analysis of F^2 against all reflections. All non-hydrogen atoms were refined with anisotropic thermal displacement parameters. Thermal parameters for hydrogen atoms were tied to the isotropic thermal parameter of the atom to which they are bonded.

Software for crystal structure analysis included Apex3 v2018.1, SHELXTL v6.14, and Diamond v4.6.1. The related details of crystallographic data and structural refinement are summed up in Table S1. The simulated powder patterns were calculated by Mercury software using the crystallographic information file from the single-crystal X-ray diffraction experiment. A full set of data was collected, however the very high angle data was dominated by noise and was omitted, leading to a level A alert.

Physical characterization. PXRD patterns were recorded using a Bruker D2 Phaser Advance diffractometer equipped with a Cu sealed tube (λ = 1.54184 Å). The diffraction patterns were scanned at 30 kV and 10 mA at ambient temperature with a speed of 0.1 sec/step, a step size of 0.02° in 2θ, and a 2θ range of 5~40°. Optical microscope images were collected by a Nikon Eclipse LV100NPOL. Fourier transform infrared spectra were collected using a Bruker Alpha spectrophotometer with a wavenumber region of 4000~400 cm^{-1}. Elemental analysis for C/H/N was measured on a Varian ELIII element analyzer. Thermogravimetric analysis (TGA) was performed using a PerkinElmer STA 8000 differential thermal analyzer. The samples were heated in N_2 atmosphere (60 mL/min) from room temperature to 800 °C with a heating rate of 10 °C/min. The emission spectra of samples were measured on a Horiba Fluorolog-3 in a reflection geometry setup. Time-resolved photoluminescence decay studies were performed at room temperature with the time correlated single time correlated single photon counting (TCSPC) technique on Horiba Fluorolog-3. The excitation wavelength was 340 nm provided by an EPL-360PS pulsed diode laser. The lifetime was calculated by fitting the data to an exponential decay function using fluorescence decay analysis software. SPV measurements were performed on the basis of a lock-in amplifier (Sr830-DSP). The measurement system includes a computer, a light chopper (SR540), monochromatic light, and a sample cell. The monochromatic light was generated by a 500 W Xenon lamp (CHFXQ500 W, Global Xenon Lamp Power) with a grating monochromator (Omni-3007, No.16047, Zolix). Ultrafast femtosecond TA spectroscopy were obtained on a Helios pumpprobe system (ultrafast systems LLC)

combined with an amplified femtosecond laser system (coherent). Metallic element content detection for Pb^{2+} was performed on a Perkin Elmer Optima 8300 ICP-OES.

Ultraviolet-Visible (UV-vis) Diffuse Reflectance Spectroscopy. UV-Vis diffuse reflectance spectrum in 200~800 nm region were recorded at room temperature upon a Shimadzu UV-2600 spectrometer equipped with integrating sphere. $BaSO_4$ was used as a reference for 100% reflectance for all measurements. Reflectance spectra were converted to absorption according to the equation:

$$A = 2\text{-lg}(T\%)$$

where A and T represent the absorbance and reflectance, respectively. The bandgap value of samples were acquired by extrapolation of the linear region of Tauc-plot.

Electrochemical Measurement. Electrochemical studies were conducted in a CHI 760E electrochemical work station (Shanghai Chenhua) in a standard three-electrode system with an indium tin oxide (ITO) deposited with TJU-26 or (FMTMA)PbI_3, a Ag/AgCl (KCl saturated) electrode and a Pt electrode as the working electrode, reference electrode and counter electrode, respectively. A 0.5M Na_2SO_4 aqueous solution was used as the electrolyte. The working electrode was prepared as follows: 50 mg of the photocatalysts were dispersed in a solution that contained H_2O (1 mL), EtOH (1 mL) and 5 wt% Nafion solution (20 μL), followed by ultrasonication for 30 min. Then, 100 μL of the above solution was drop cast onto the surface of an ITO plate (1.0 cm × 2.0 cm) and dried at room temperature.

AC Hall measurement. Hall effect measurements were performed on the Accent HL5500 Hall System, which samples are deposited on the glass substrate with four-point gold electrode contacts on each corner according to van der Pauw technique. The magnetic field for the test is 0.5 T and the range of test current is from -200 to 200 mA. The Hall system use a rotating parallel dipole line magnet that generates AC field with

pure harmonic, unidirectional, and strong magnetic field followed by Fourier spectral analysis and lock-in detection of the Hall signal.

Photocatalytic CO_2 Reduction. 20 mg of the photocatalyst was dispersed in 10 mL absolute ethanol, which was added to a Perfect Light top-irradiation-type reaction cell. The reaction system was vacuum-treated and refilled with high-purity CO_2 (99.99 %) for three times to ensure all the impurities and trapped air were completely removed, and was refilled with CO_2 to 1 atm. The temperature of the reaction cell was controlled at 4 °C by recirculating cooling water system during irradiation. The light source for the photocatalysis was a 300 W Xe lamp (PLS-SXE300/300UV, Beijing Perfect light Technology Co., Ltd.). Upon the light irradiation, the product gases were qualitatively analyzed by the online headspace gas chromatograph (GC7860Plus, Shanghai Nuoxi Instrument Co., Ltd.) with a flame ionization detector (FID) and for CO and CH_4 determinations by identifying the chromatographic peaks. The liquid product was diluted and dispersed in D_2O, followed by ^{1}H nuclear magnetic resonance spectroscopy analysis (Advance III HD spectrometer, 600 MHz) using 100 μL diluted dimethyl sulfoxide (DMSO) in water (0.056 μM) as the internal standard. The cyclic photocatalytic test was conducted as follows: after each reaction cycle, the used photocatalyst was recovered by centrifugation and reused in the subsequent test. This process was repeated for a total of four cycles.

In situ diffuse reflectance infrared Fourier transform spectroscopy (In situ DRIFTS). In situ DRIFTS of TJU-26 in photocatalysis was performed on a Thermo Scientific Nicolet 6700FT spectrometer. The CO_2 flow was bubbled into ethanol and then passed through TJU-26 powders which loaded on the center of sample cell. The Xenon lamp light was irradiated on the sample through a quartz window of the sample cell. The gaseous mixture of CO_2 and EtOH was kept steady before the light irradiation. After then, the DRIFT signals were collected in situ through the MCT detector.

AQE measurement for photocatalytic CO_2 reduction. The AQEs of CO_2-to-CH_3OH conversion were measured under specific excitation wavelength with a band pass filter

(λ = 400, 425, 500, 525 nm), which was irradiated by the 300 W Xe lamp on the same setup. The number of the incident photons was measured by using a radiant power energy meter (PL-MW2000 Photoradiometer, Perfect Light Co., Ltd.). In general, the AQE of CO_2-to-CH_3OH conversion is calculated as follows:

$$\mathrm{AQE}\ (\%) = \frac{N_{\mathrm{MeOH}} \times 6 \times N_{\mathrm{A}}}{H_a \times A \times \frac{\lambda}{hc} \times t} \times 100\% \tag{1}$$

where N_{MeOH} is the amount of CH_3OH after 4 h reaction, N_{A} is the Avogadro's number, Ha is the average intensity of absorbed light (42 mW/cm^{-2}), obtained by the subtraction of the transmitted intensity from the incident intensity. A is the irradiation area (0.64 cm^2), h is the Planck's constant, c is the speed of light, λ is the wavelength of the incident light, t is the time.

Vapor adsorption isotherms. TJU-26 (ca. 100 mg) were transferred in a pre-weighed analysis tube, heated at 80 °C under the outgas rate to < 5 mm Hg for 600 min to remove all residual solvents on the Micromeritics ASAP 2020 absorption analyzer. The sample tube was re-weighed to obtain a consistent dry mass for the degassed sample. Vapor sorption isotherms were recorded volumetrically at 298 K for EtOH and CH_3OH, respectively.

Computational Details. Density functional theory (DFT) calculations were performed by using the Vienna ab initio Simulation Program (VASP). The generalized gradient approximation (GGA) in the Perdew-Burke-Ernzerhof (PBE) form and a cutoff energy of 500 eV for planewave basis set were adopted. A 2 × 2 × 1 Monkhorst-Pack grid was used for sampling the Brillouin zones at structure optimization. The ion-electron interactions were described by the projector augmented wave (PAW) method. The convergence criteria of structure optimization were choose as the maximum force on each atom less than 0.02 eV/Å with an energy change less than 1 × 10^{-5} eV. The DFT-D3 semiempirical correction was described via Grimme's scheme method. The standard hydrogen electrode model proposed by Nørskov and co-workers was

employed to calculate the Gibbs free-energy change (ΔG) for each elemental step. The ΔG is defined as:

$$\Delta G = \Delta E + \Delta ZPE - T\Delta S + \Delta G_{\mathrm{U}} + \Delta G_{\mathrm{pH}} \quad (2)$$

where ΔE and ΔZPE are the adsorption energy based on density functional theory calculations and the zero-point energy correction, respectively. T, ΔS, U, and ΔG_{pH} represent the temperature, the entropy change, the applied electrode potential, and the free energy correction of the pH, respectively.

Table S1. Crystal data and structure refinement of TJU-26.

Empirical formula	$[Pb_2I_2]^{2+}[PbO][Fcdc^{2-}]$
Formula weight	1163.40
Temperature/K	297.00
Crystal system	orthorhombic
Space group	*Cmce*
a/Å	18.3688(7)
b/Å	10.6562(7)
c/Å	21.9752(8)
α/°	90
β/°	90
γ/°	90
Volume/Å^3	4301.5(4)
Z	8
ρ_{calc} g/cm^3	3.593
μ/mm^{-1}	26.959
F(000)	3984.0
Crystal size/mm^3	0.12×0.11×0.11
Radiation	MoKα (λ = 0.71073)
2θ range for data collection/°	5.77 to 42.552
Index ranges	$-18 \leq h \leq 18$, $-10 \leq k \leq 10$, $-22 \leq l \leq 22$
Reflections collected	26419
Independent reflections	1226 [R_{int} = 0.1154, R_{sigma} = 0.0369]
Data/restraints/parameters	1226/137/124
Goodness-of-fit on F^2	1.116
Final R indexes [I>=2σ (I)]	R_1 = 0.1395, wR_2 = 0.3051
Final R indexes [all data]	R_1 = 0.1418, wR_2 = 0.3058
Largest diff. peak/hole/e Å^{-3}	2.92/-2.99

$R_1=\sum(||F_0|-|F_c||) / \sum|F_0|$; $wR_2=\{\sum[w(F_0^2-F_c^2)]/\sum[w(F_0^2)]^2\}^{1/2}$

Table S2. Recent literature summary of the photocatalytic CO_2 reduction performances based on lead halide hybrids. Notably, no photocatalytic CO_2 to CH_3OH transformation has been observed in this class of organolead halide hybrids.

Photocatalyst	Solvents	light source	Products (μmol/g/h)	Ref.
$CsPbBr_3$ QDs	ethyl acetate / water	300 W Xe lamp (AM1.5G)	CO (4.3), CH_4 (1.5)	S2
$CsPbBr_3$ QD/GO	ethyl acetate	100 W Xe lamp (AM1.5G)	CO (4.9), CH_4 (2.5)	S3
$CsPbBr_3$QDs/g-C_3N_4	acetonitrile / water	300 W Xe lamp (420 nm cutoff filter)	CO (148.9)	S4
$CsPbBr_3$/TiO-g-C_3N_4	acetonitrile / water	300 W Xe lamp (400 nm cutoff filter)	CO (12.9)	S5
$CsPbBr_3$/MXene	ethyl acetate	300 W Xe lamp (420 nm cutoff filter)	CO (26.3), CH_4 (7.3)	S6
$CsPb(Br_{0.5}Cl_{0.5})_3$ QDs	ethyl acetate	300 W Xe lamp (AM1.5G)	CO (85.2), CH_4 (12.0)	S7
$CsPbBr_3$ NC/a-TiO_2(20)	ethyl acetate / isopropanol	150 W Xe lamp (AM1.5G)	CO (3.9), CH_4 (6.72)	S8
$MAPbI_3$@PCN-221($Fe_{0.2}$)	ethyl acetate / water	300 W Xe lamp (400 nm cutoff filter)	CO (4.16), CH_4 (13)	S9
$Co_{2\%}$@$CsPbBr_3$/Cs_4PbBr_6	water	Xe lamp (400 nm cutoff filter)	CO (11.9)	S10

Table S3. A literature summary of the photocatalytic CO_2-to-CH_3OH transformation by various photocatalysts.

Photocatalyst	solution	light source	CH_3OH (μmol/g/h)	Selectivity	Ref.
Single unit cell Bi_2WO_6 layers	water	300 W Xe lamp (AM1.5G)	75.0	n.d.	S11
PTh/Bi_2WO_6	water	300 W Xe lamp (420 nm cutoff filter)	14.1	73.4%	S12
V-$Bi_{19}Br_3S_{27}$	water	300 W Xe lamp (420 nm cutoff filter)	0.6	n.d.	S13
^{m}CD/CN	water	300 W Xe lamp	13.9	99.6%	S14
rGO–CuO116	DMF/water	Visible light	51.2	n.d.	S15
OCN-Tube	Water vapor	350 W Xe lamp (420 nm cutoff filter)	0.9	n.d.	S16
Bi_2S_3/ TiO_2 nanotube	water	500 W Xe lamp	44.9	n.d.	S17
Carbon/TiO_2 hollow spheres	Water vapor	300 W Xe lamp ($\lambda >$ 200 nm)	9.1	n.d.	S18
NiO (1.0 wt%)/ $InTaO_4$	Water/$KHCO_3$	500 W halogen lamp	1.4	n.d.	S19

n.d=not determined

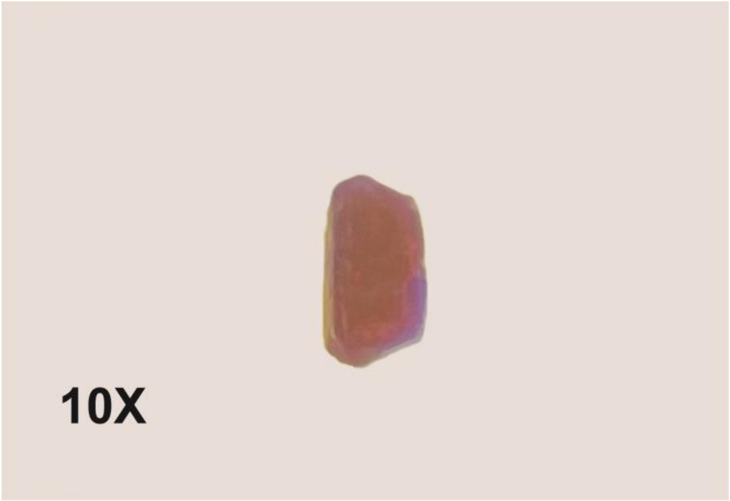

Figure S1. Optical image of a single crystal of TJU-26.

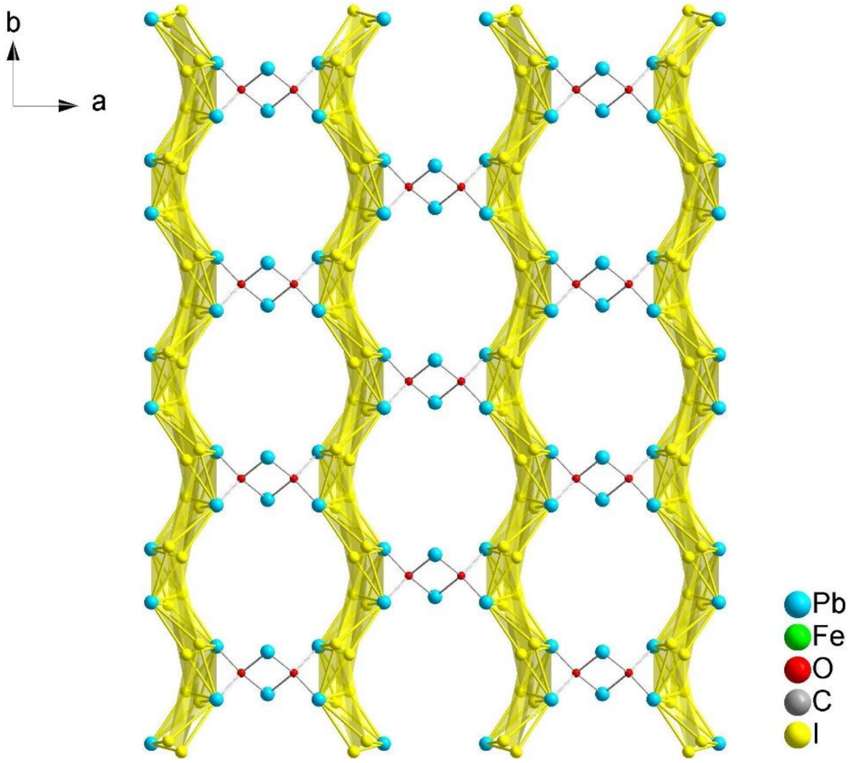

Figure S2. Crystallographic view of the inorganic sublattice of TJU-26 along the *c*-axis.

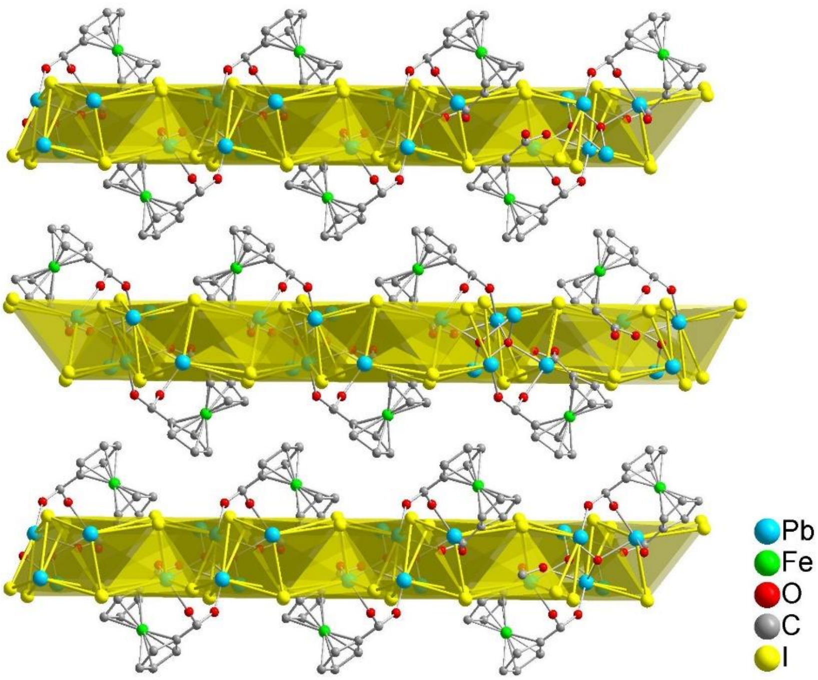

Figure S3. Crystallographic view of TJU-26 along the *a*-axis.

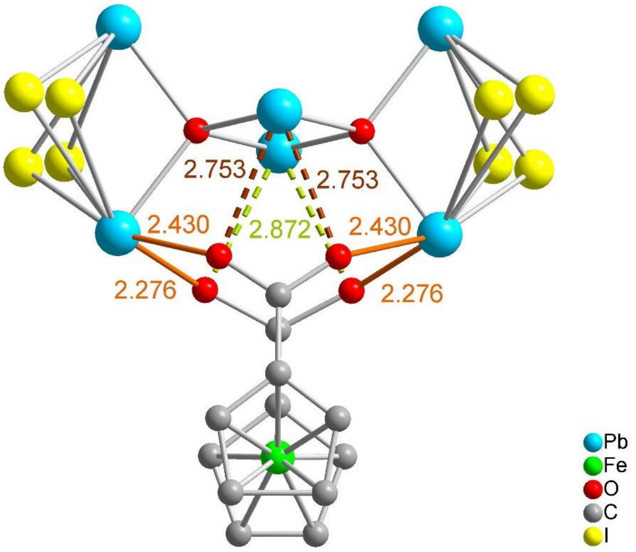

Figure S4. The Pb-O bond distances between the Pb^{2+} centers and the carboxylate oxygens in TJU-26. The Pb-O bond distances between the $[Pb_2I_2]^{2+}$ chains and $Fcdc^{2-}$ ligands are 2.276~2.430 Å. The Pb-O bond distances between the neutral $[Pb_2O_2]$ units and $Fcdc^{2-}$ ligands are 2.753~2.872 Å.

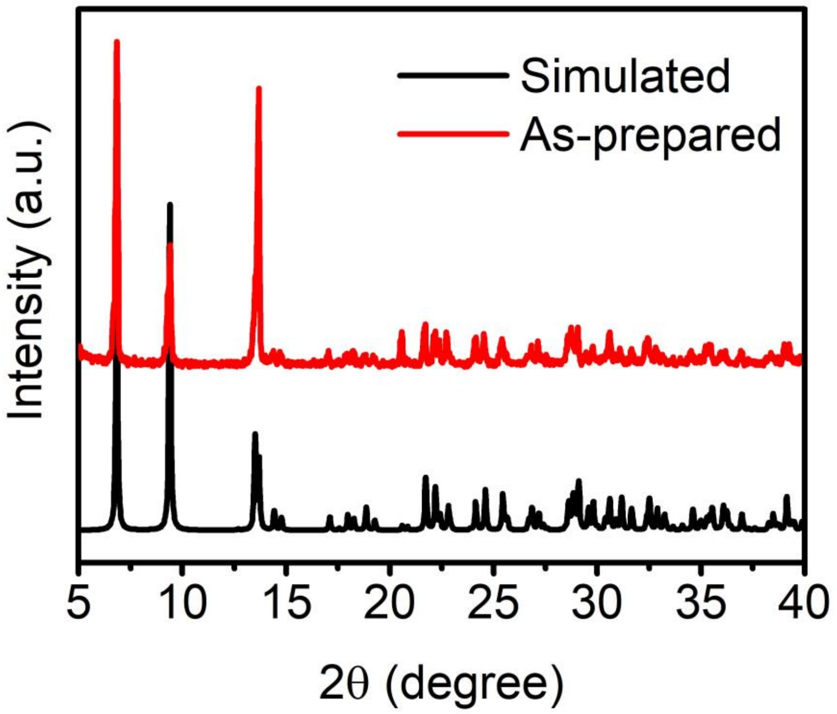

Figure S5. PXRD of (FMTMA)PbI_3.

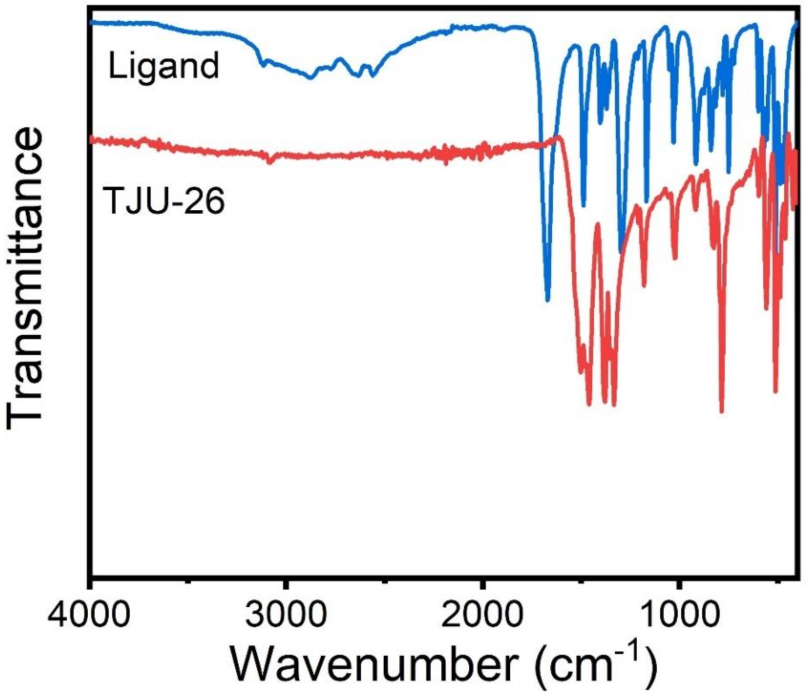

Figure S6. FT-IR spectra of H_2Fcdc and TJU-26.

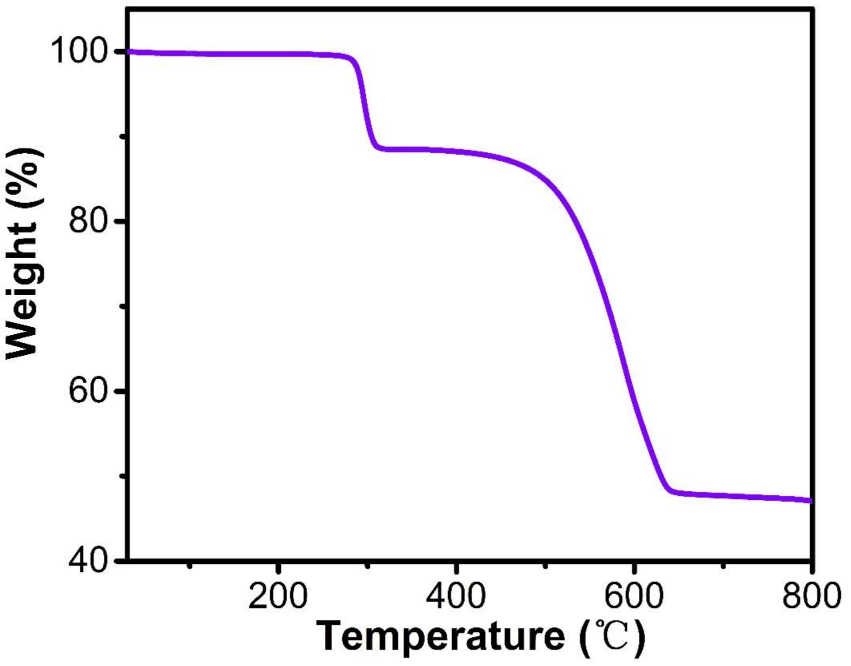

Figure S7. TGA of TJU-26 in N_2 flow.

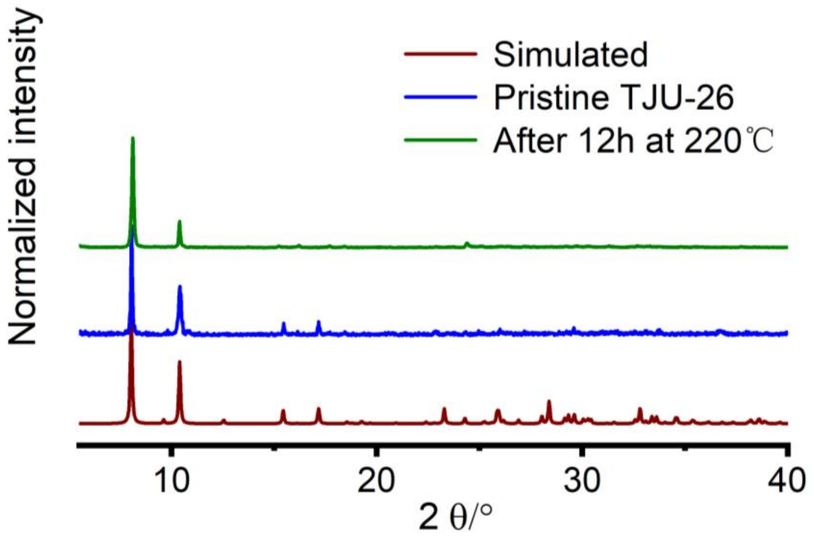

Figure S8. PXRD of TJU-26 before and after thermal treatment. TJU-26 was heated at 220°C in air for 12 h before PXRD characterization.

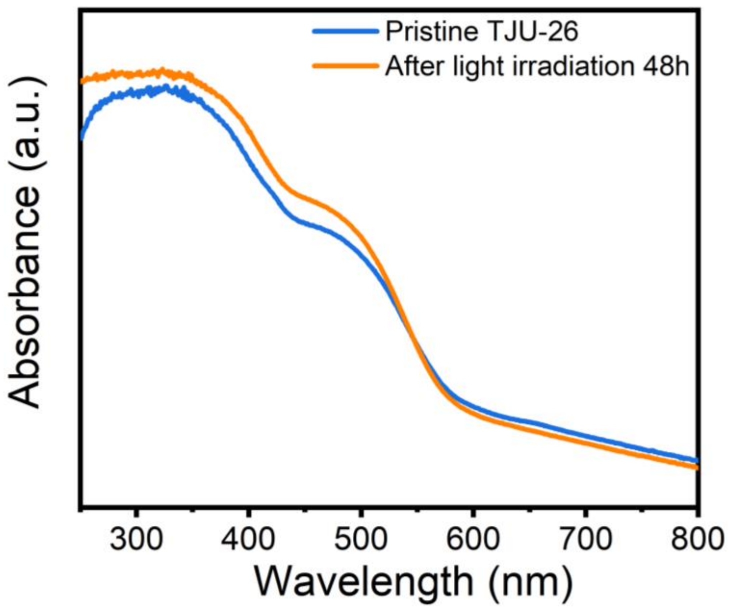

Figure S9. UV–vis diffuse reflectance spectroscopy of TJU-26 before and after light irradiation (300 W Xe lamp, AM 1.5G) for 48 h.

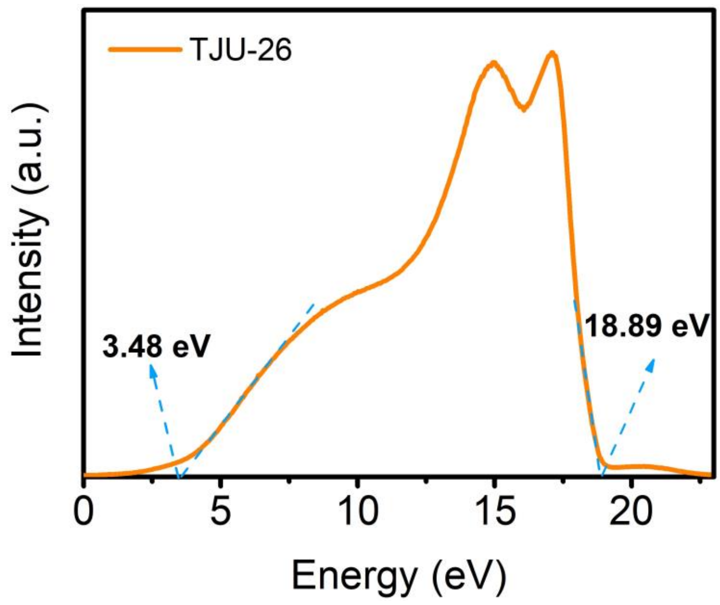

Figure S10. Ultraviolet photoelectron spectroscopy of TJU-26.

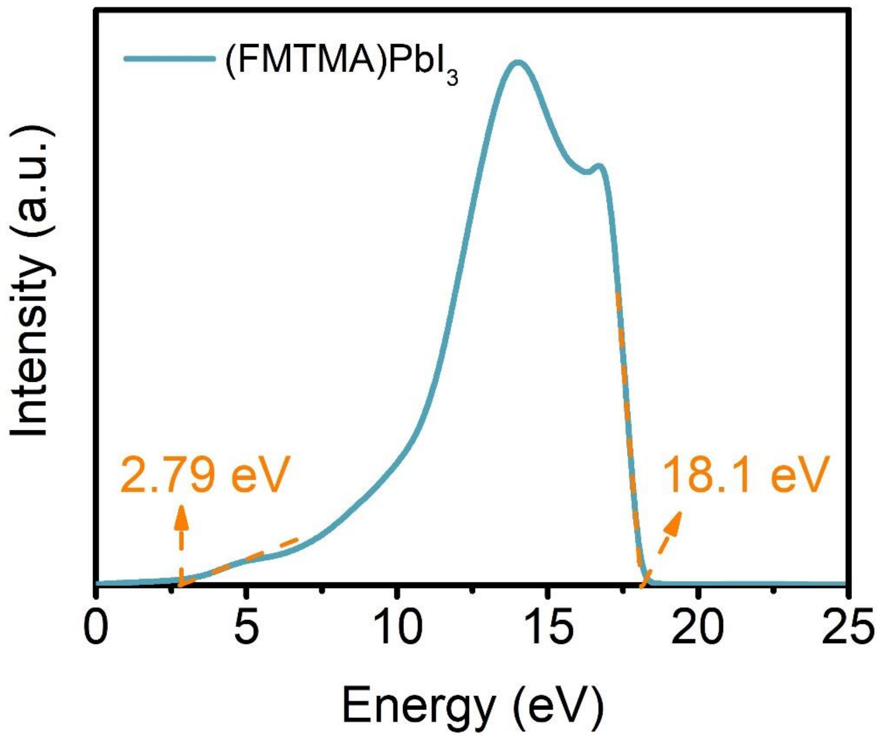

Figure S11. Ultraviolet photoelectron spectroscopy of (FMTMA)PbI_3.

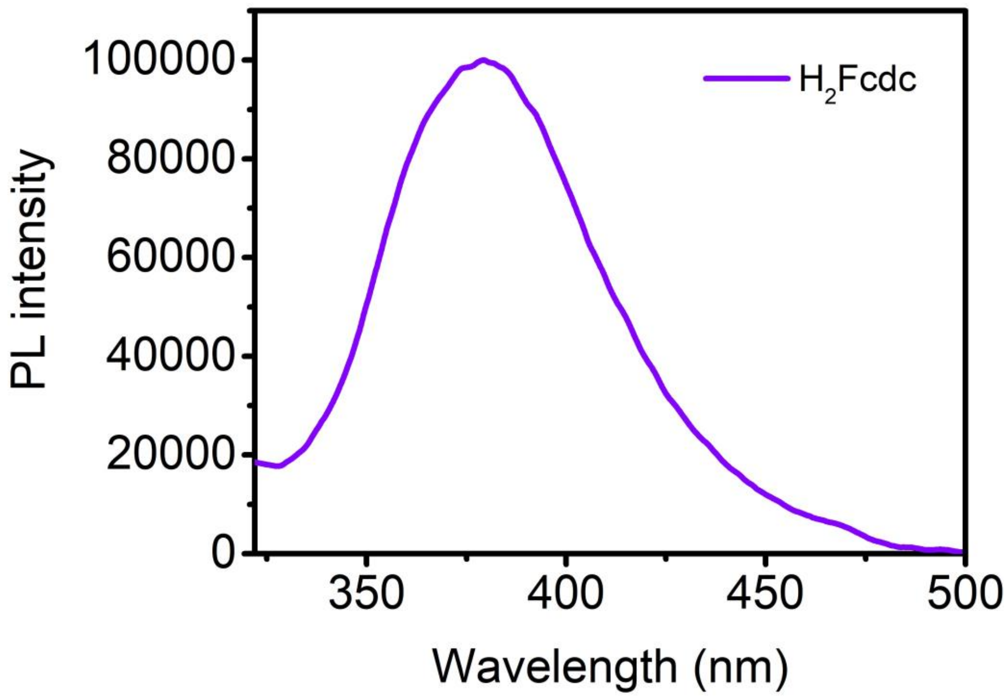

Figure S12. Emission spectra of H_2Fcdc at room temperature (ex. 340 nm).

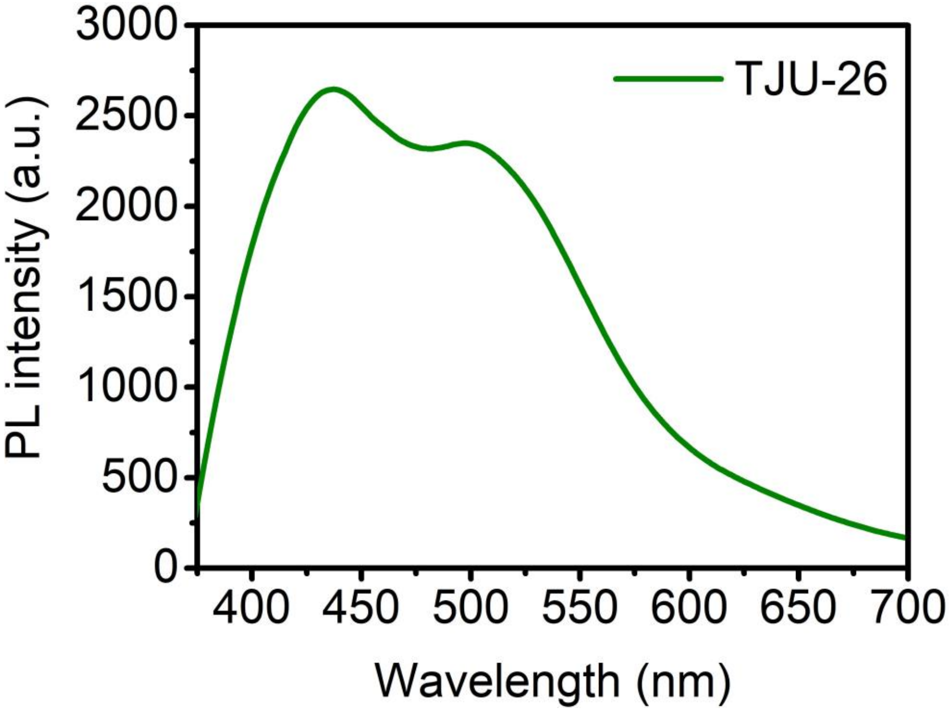

Figure S13. Emission spectra of TJU-26 at room temperature (ex. 355 nm).

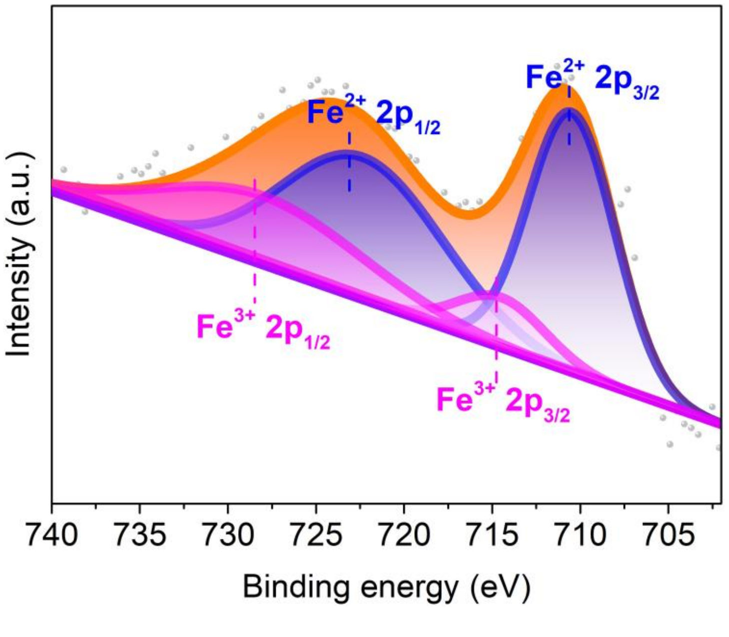

Figure S14. XPS spectra of Fe 2*p* of TJU-26.

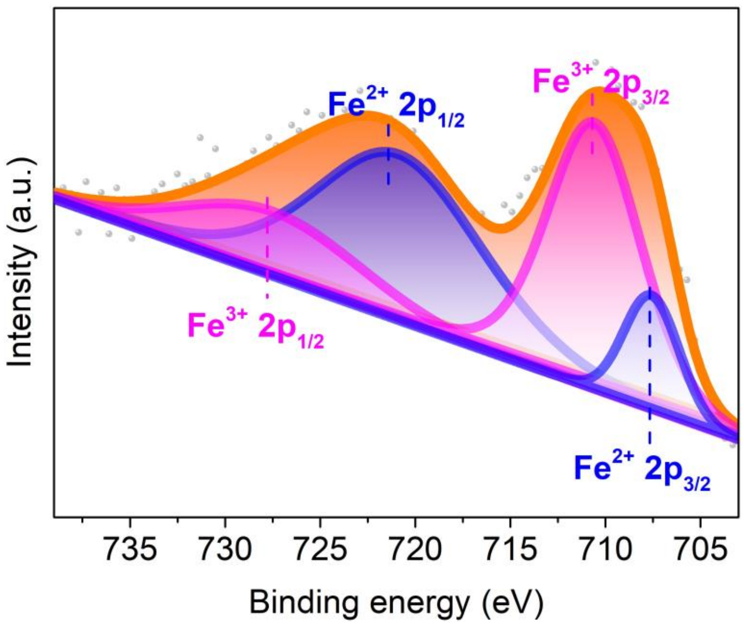

Figure S15. XPS spectra of Fe 2*p* in TJU-26 photocatalyst under 300W Xe lamp irradiation.

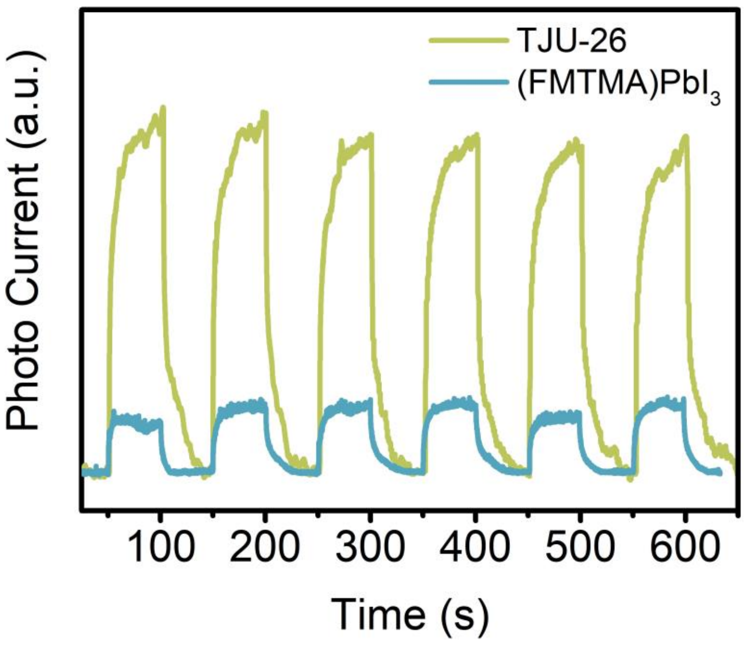

Figure S16. Photocurrent measurements for TJU-26 and (FMTMA)PbI_3 under AM 1.5G irradiation in the aqueous solution of 0.5 M Na_2SO_4.

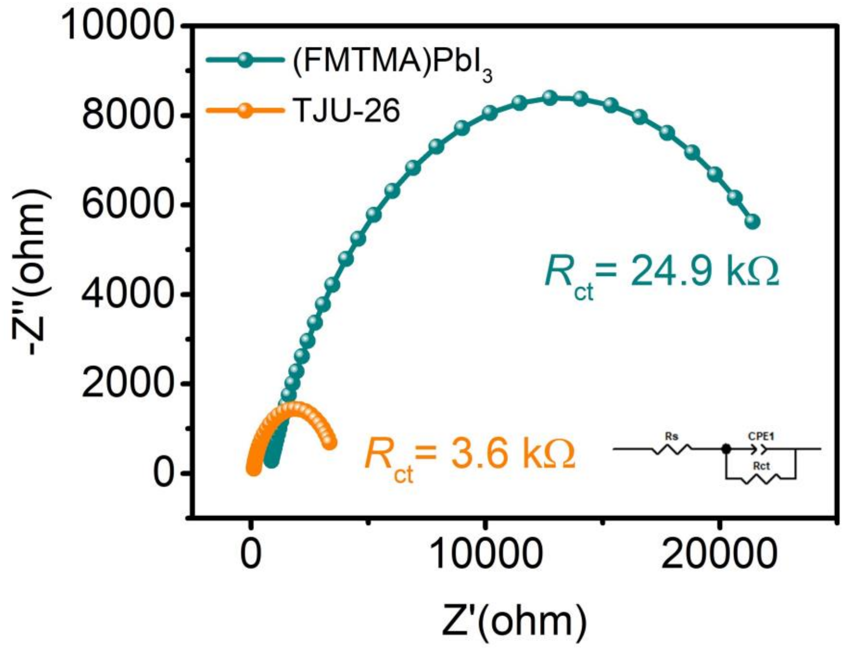

Figure S17. EIS plots of TJU-26 and (FMTMA)PbI_3.

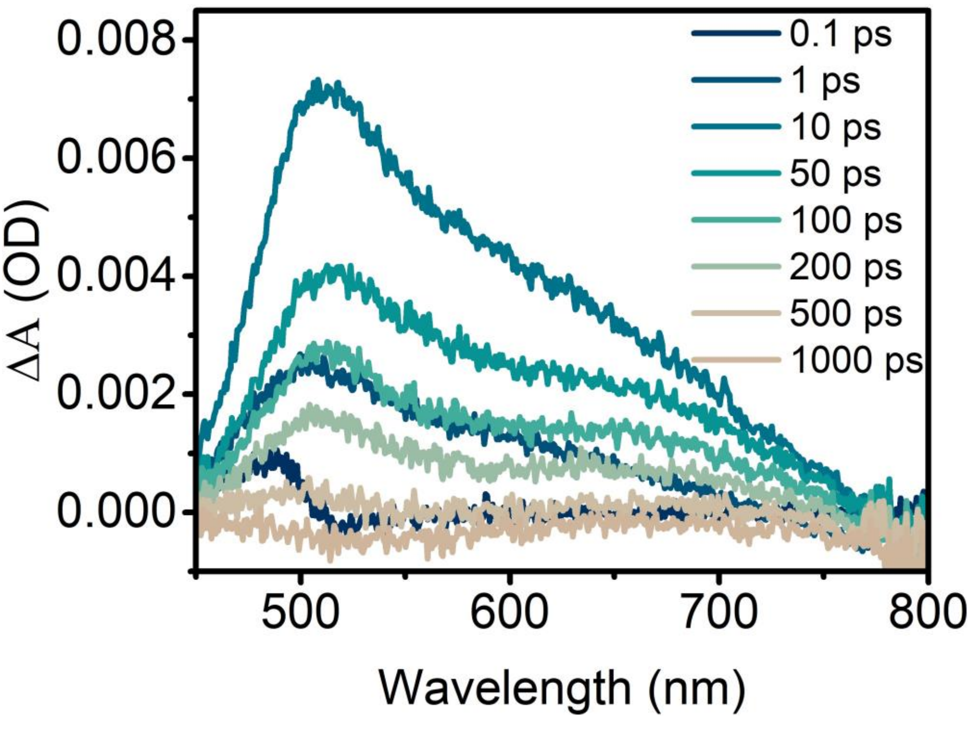

Figure S18. TA spectra of TJU-26 measured at different delay times.

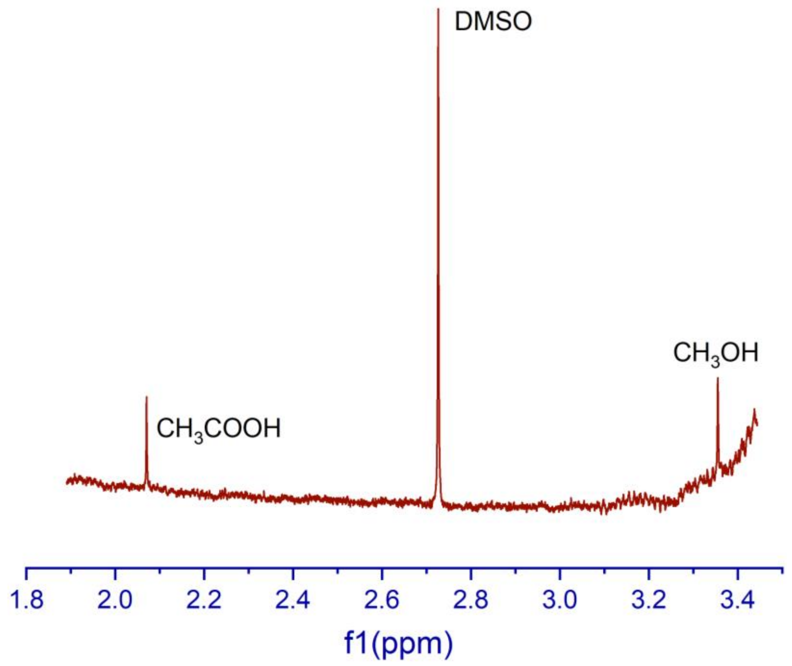

Figure S19. ^{1}H NMR of the liquid products obtained by reduction of CO_2 with TJU-26 for 4 h in the visible light.

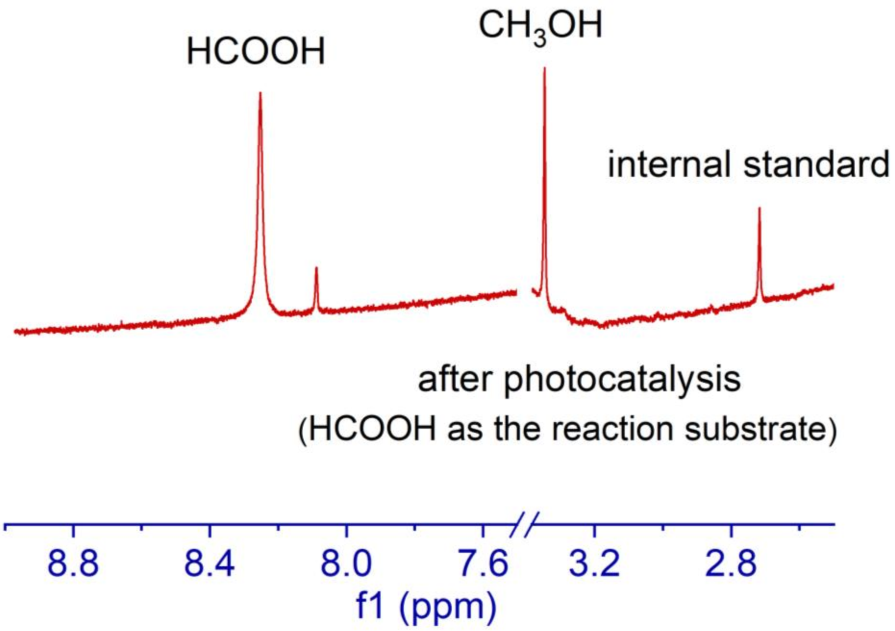

Figure S20. ^{1}H NMR of the liquid products obtained by reduction of HCOOH with TJU-26 for 4 h in the visible light.

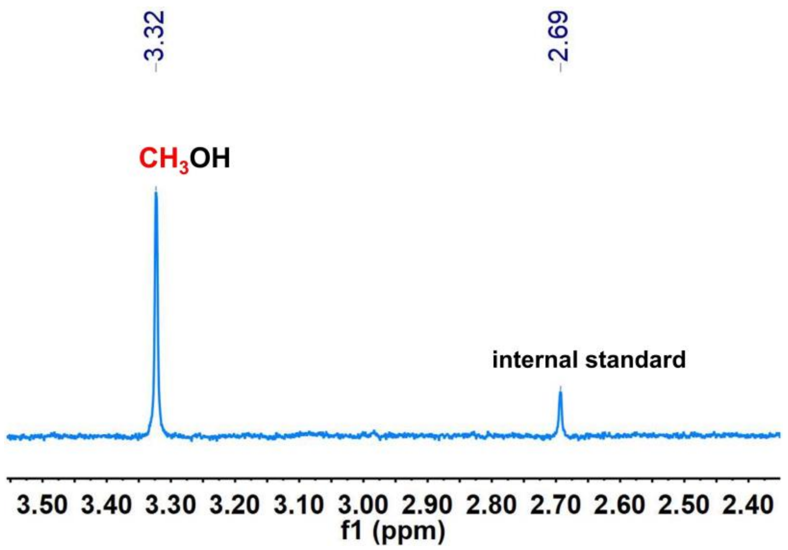

Figure S21. ^{1}H NMR of the liquid product obtained after 4 h CO_2 photoreduction by TJU-26 in H_2O with sodium sulfite as the hole scavenger.

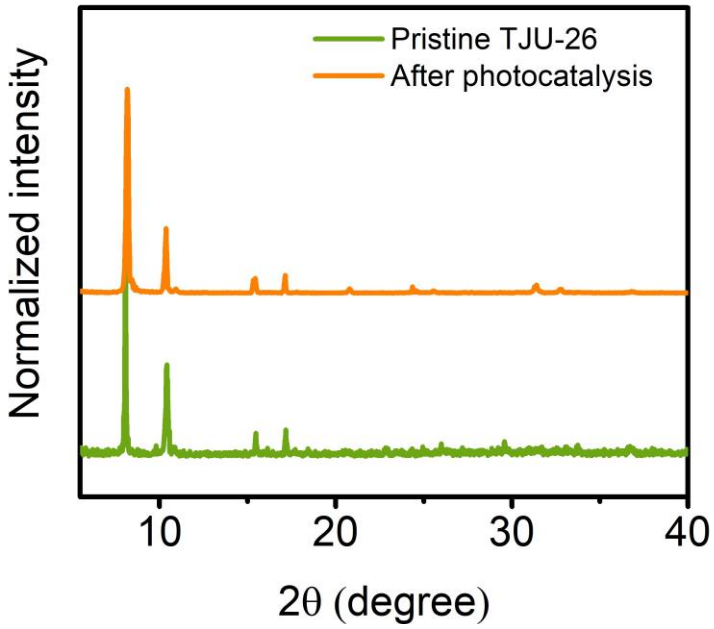

Figure S22. PXRD of TJU-26 before and after four photocatalytic cycles.

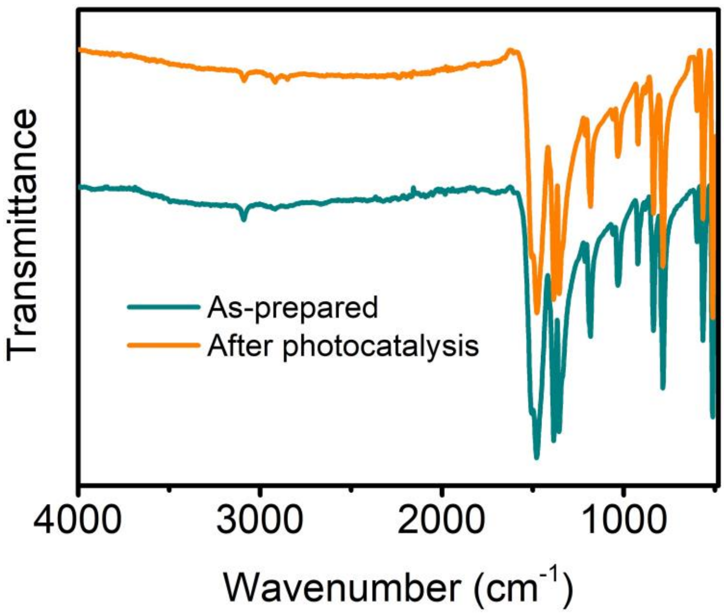

Figure S23. FT-IR spectra of TJU-26 before and after four photocatalytic cycles.

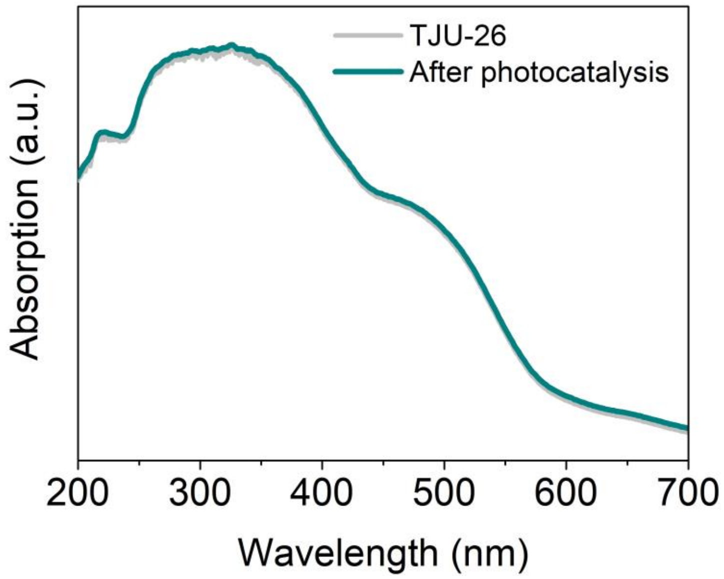

Figure S24. UV–vis diffuse reflectance spectroscopy of TJU-26 before and after four photocatalytic cycles.

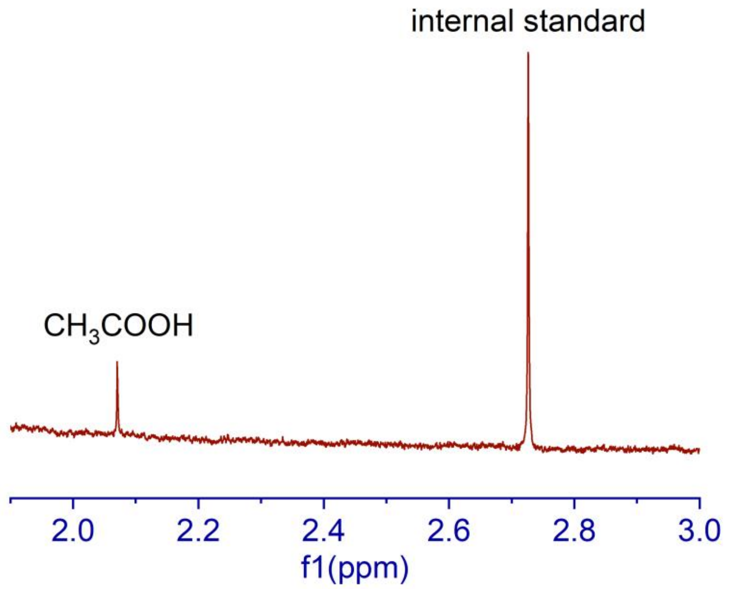

Figure S25. ^{1}H-NMR spectra of photocatalytic EtOH oxidation products using TJU-26 as catalysts under simulated sunlight for 12 h.

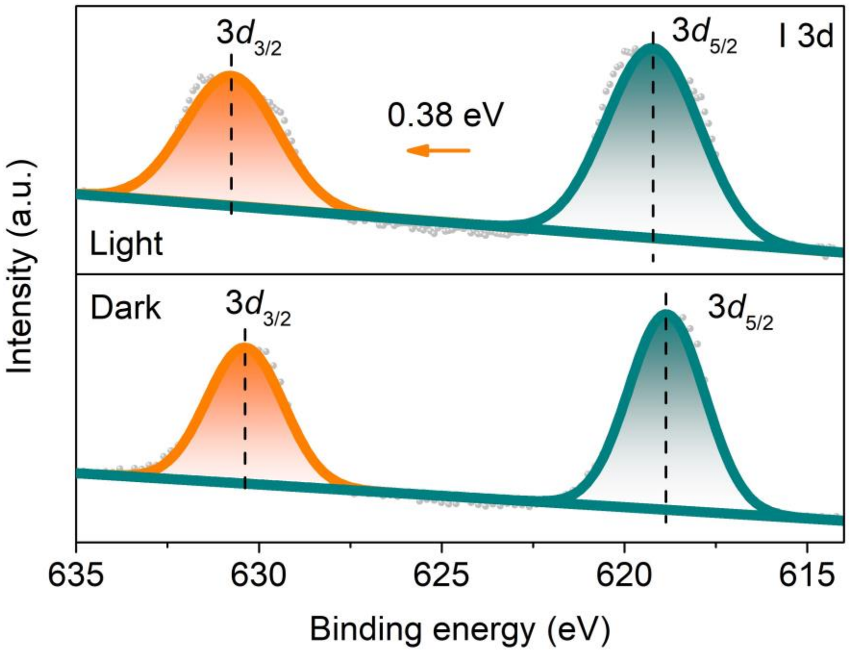

Figure S26. Quasi in situ XPS spectra before and after light irradiation of I 3*d* of TJU-26.

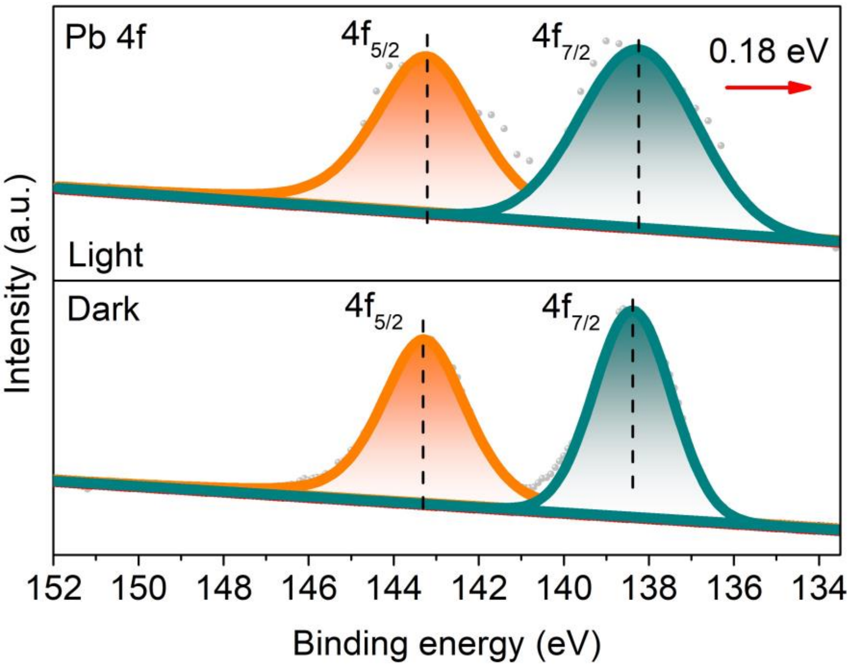

Figure S27. Quasi in situ XPS spectra before and after light irradiation of Pb 4*f* of TJU-26.

Supporting Information References

[S1] Zhang, Z.-X.; Zhang, H.-Y.; Zhang, W.; Chen, X.-G.; Wang, H.; Xiong, R.-G. Organometallic-based hybrid perovskite piezoelectrics with a narrow band gap. *J. Am. Chem. Soc.* **2020**, *142*, 17787-17794.

[S2] Hou, J.; Cao, S.; Wu, Y.; Gao, Z.; Liang, F.; Sun, Y.; Lin, Z.; Sun, L., Inorganic colloidal perovskite quantum dots for robust solar CO_2 reduction. *Chem. Eur. J.* **2017,** *23*, 9481-9485.

[S3] Xu, Y. F.; Yang, M. Z.; Chen, B. X.; Wang, X. D.; Chen, H. Y.; Kuang, D. B.; Su, C. Y., A $CsPbBr_3$ perovskite quantum dot/graphene oxide composite for photocatalytic CO_2 reduction. *J. Am. Chem. Soc.* **2017,** *139*, 5660-5663.

[S4] Ou, M.; Tu, W.; Yin, S.; Xing, W.; Wu, S.; Wang, H.; Wan, S.; Zhong, Q.; Xu, R., Amino-assisted anchoring of $CsPbBr_3$ perovskite quantum dots on porous g-C_3N_4 for enhanced photocatalytic CO_2 reduction. *Angew. Chem.* **2018,** *130*, 13758-13762.

[S5] Guo, X. X.; Tang, S. F.; Mu, Y. F.; Wu, L. Y.; Dong, G. X.; Zhang, M., Engineering a $CsPbBr_3$-based nanocomposite for efficient photocatalytic CO_2 reduction: improved charge separation concomitant with increased activity sites. *RSC Adv.* **2019,** *9*, 34342-34348.

[S6] Pan, A.; Ma, X.; Huang, S.; Wu, Y.; Jia, M.; Shi, Y.; Liu, Y.; Wangyang, P.; He, L.; Liu, Y., $CsPbBr_3$ perovskite nanocrystal grown on MXene nanosheets for enhanced photoelectric detection and photocatalytic CO_2 reduction. *J. Phys. Chem. Lett.* **2019,** *10*, 6590-6597.

[S7] Guo, S. H.; Zhou, J.; Zhao, X.; Sun, C.-Y.; You, S. Q.; Wang, X. L.; Su, Z. M., Enhanced CO_2 photoreduction via tuning halides in perovskites. *J. Catal.* **2019,** *369*, 201-208.

[S8] Xu, Y. F.; Wang, X. D.; Liao, J. F.; Chen, B. X.; Chen, H. Y.; Kuang, D. B., Amorphous-TiO_2-encapsulated $CsPbBr_3$ nanocrystal composite photocatalyst with enhanced charge separation and CO_2 fixation. *Adv. Mater. Interfaces.* **2018,** *5*, 1801015.

[S9] Wu, L.-Y., Mu, Y.-F., Guo, X.-X., Zhang, W., Zhang, Z.-M., Zhang, M., Lu, T.-B., Encapsulating perovskite quantum dots in iron-based metal–organic frameworks (MOFs) for efficient photocatalytic CO_2 reduction. *Angew. Chem. Int. Ed.* **2019**, *58*, 9491-9495.

[S10] Mu, Y. F.; Zhang, W.; Guo, X. X.; Dong, G. X.; Zhang, M.; Lu, T. B., Water-tolerant lead halide perovskite nanocrystals as efficient photocatalysts for visible-light-driven CO_2 reduction in pure water. *ChemSusChem.* **2019,** *12*, 4769-4774.

[S11] Liang, L.; Lei, F.; Gao, S.; Sun, Y.; Jiao, X.; Wu, J.; Qamar, S.; Xie, Y., Single unit cell bismuth tungstate layers realizing robust solar CO_2 reduction to methanol. *Angew. Chem. Int. Ed.* **2015,** *54*, 13971-13974.

[S12] Dai, W.; Xu, H.; Yu, J.; Hu, X.; Luo, X.; Tu, X.; Yang, L., Photocatalytic reduction of CO_2 into methanol and ethanol over conducting polymers modified Bi_2WO_6 microspheres under visible light. *Appl. Surf. Sci.* **2015,** *356*, 173-180.

[S13] Li, J.; Pan, W.; Liu, Q.; Chen, Z.; Chen, Z.; Feng, X.; Chen, H., Interfacial engineering of $Bi_{19}Br_3S_{27}$ nanowires promotes metallic photocatalytic CO_2 reduction activity under near-infrared light irradiation. *J. Am. Chem. Soc.* **2021,** *143*, 6551-6559.

[S14] Wang, Y.; Liu, X.; Han, X.; Godin, R.; Chen, J.; Zhou, W.; Jiang, C.; Thompson, J. F.; Mustafa, K. B.; Shevlin, S. A.; Durrant, J. R.; Guo, Z.; Tang, J., Unique hole-accepting carbon-dots promoting selective carbon dioxide reduction nearly 100% to methanol by pure water. *Nat. Commun.* **2020,** *11*, 2531.
[S15] Gusain, R.; Kumar, P.; Sharma, O. P.; Jain, S. L.; Khatri, O. P., Reduced graphene oxide-CuO nanocomposites for photocatalytic conversion of CO_2 into methanol under visible light irradiation. *Appl. Catal., B.* **2016,** *181,* 352-362.
[S16] Fu, J.; Zhu, B.; Jiang, C.; Cheng, B.; You, W.; Yu, J., Hierarchical porous O-doped g-C_3N_4 with enhanced photocatalytic CO_2 reduction activity. *Small* **2017,** *13,* 1603938.
[S17] Li, X.; Liu, H.; Luo, D.; Li, J.; Huang, Y.; Li, H.; Fang, Y.; Xu, Y.; Zhu, L., Adsorption of CO_2 on heterostructure $CdS(Bi_2S_3)/TiO_2$ nanotube photocatalysts and their photocatalytic activities in the reduction of CO_2 to methanol under visible light irradiation. *Chem. Eng. J.* **2012,** *180*, 151-158.
[S18] Wang, W.; Xu, D.; Cheng, B.; Yu, J.; Jiang, C., Hybrid carbon@TiO_2 hollow spheres with enhanced photocatalytic CO_2 reduction activity. *J. Mater. Chem. A.* **2017,** *5,* 5020-5029.
[S19] Pan, P. W.; Chen, Y. W., Photocatalytic reduction of carbon dioxide on $NiO/InTaO_4$ under visible light irradiation. *Catal. Commun.* **2007,** *8,* 1546-1549.
